# Structure and ion-release mechanism of P_IB-4_-type ATPases

**DOI:** 10.7554/eLife.73124

**Published:** 2021-12-24

**Authors:** Christina Grønberg, Qiaoxia Hu, Dhani Ram Mahato, Elena Longhin, Nina Salustros, Annette Duelli, Pin Lyu, Viktoria Bågenholm, Jonas Eriksson, Komal Umashankar Rao, Domhnall Iain Henderson, Gabriele Meloni, Magnus Andersson, Tristan Croll, Gabriela Godaly, Kaituo Wang, Pontus Gourdon

**Affiliations:** 1 Department of Biomedical Sciences, University of Copenhagen Copenhagen Denmark; 2 Department of Chemistry, Umeå University Umeå Sweden; 3 Department of Sciences, University of Copenhagen Copenhagen Denmark; 4 Department of Laboratory Medicine, Lund University Lund Sweden; 5 Department of Chemistry and Biochemistry, The University of Texas Dallas United States; 6 Cambridge Institute for Medical Research, Department of Haematology, University of Cambridge Cambridge United Kingdom; 7 Department of Experimental Medical Science, Lund University Lund Sweden; Stanford University School of Medicine United States; Weill Cornell Medicine United States

**Keywords:** P-type ATPase, x-ray crystallography, sulfitobacter sp. NAS14-1, transition metals, PIB-4-ATPase, Other

## Abstract

Transition metals, such as zinc, are essential micronutrients in all organisms, but also highly toxic in excessive amounts. Heavy-metal transporting P-type (P_IB_) ATPases are crucial for homeostasis, conferring cellular detoxification and redistribution through transport of these ions across cellular membranes. No structural information is available for the P_IB-4_-ATPases, the subclass with the broadest cargo scope, and hence even their topology remains elusive. Here, we present structures and complementary functional analyses of an archetypal P_IB-4_-ATPase, sCoaT from *Sulfitobacter* sp. NAS14-1. The data disclose the architecture, devoid of classical so-called heavy-metal-binding domains (HMBDs), and provide fundamentally new insights into the mechanism and diversity of heavy-metal transporters. We reveal several novel P-type ATPase features, including a dual role in heavy-metal release and as an internal counter ion of an invariant histidine. We also establish that the turnover of P_IB_-ATPases is potassium independent, contrasting to many other P-type ATPases. Combined with new inhibitory compounds, our results open up for efforts in for example drug discovery, since P_IB-4_-ATPases function as virulence factors in many pathogens.

## eLife digest

Heavy metals such as zinc and cobalt are toxic at high levels, yet most organisms need tiny amounts for their cells to work properly. As a result, proteins studded through the cell membrane act as gatekeepers to finetune import and export. These proteins are central to health and disease; their defect can lead to fatal illnesses in humans, and they also help bacteria infect other organisms.

Despite their importance, little is known about some of these metal-export proteins. This is particularly the case for P_IB-4_-ATPases, a subclass found in plants and bacteria and which includes, for example, a metal transporter required for bacteria to cause tuberculosis. Intricate knowledge of the three-dimensional structure of these proteins would help to understand how they select metals, shuttle the compounds in and out of cells, and are controlled by other cellular processes.

To reveal this three-dimensional organisation, Grønberg et al. used X-ray diffraction, where high-energy radiation is passed through crystals of protein to reveal the positions of atoms. They focused on a type of PIB-4-ATPases found in bacteria as an example.

The work showed that the protein does not contain the metal-binding regions seen in other classes of metal exporters; however, it sports unique features that are crucial for metal transport such as an adapted pathway for the transport of zinc and cobalt across the membrane. In addition, Grønberg et al. tested thousands of compounds to see if they could block the activity of the protein, identifying two that could kill bacteria.

This better understanding of how PIB-4-ATPases work could help to engineer plants capable of removing heavy metals from contaminated soils, as well as uncover new compounds to be used as antibiotics.

## Introduction

The ability to adapt to environmental changes in heavy-metal levels is paramount for all cells, as these elements are essential for a range of cellular processes and yet toxic at elevated concentrations (Waldron et al., 2009; Kozlowski et al., 2009). Transition metal transporting P-type (P_IB_) ATPase proteins are critical for cellular heavy-metal homeostasis, providing efflux of for example copper, zinc, and cobalt from the intracellular milieu. Indeed, malfunctioning of the human P_IB_-members, ATP7A and ATP7B, cause the fatal neurological Menkes disease and Wilson disease (Bull et al., 1993; Vulpe et al., 1993). The P_IB_-ATPases belong to the P-type ATPase superfamily of integral membrane proteins, which exploit energy from ATP hydrolysis for transport of cargo across cellular membranes. These proteins share an overall mechanism described by the so-called Post-Albers cycle (Albers et al., 1963; Post and Sen, 1965), as established by decades of structural and functional investigations of primarily Ca^2+^-, Na^+^/K^+^-, and H^+^-specific P-type ATPases (Toyoshima et al., 2000; Toyoshima and Nomura, 2002; Toyoshima et al., 2004; Olesen et al., 2004; Olesen et al., 2007; Winther et al., 2013; Toyoshima et al., 2013; Morth et al., 2007; Shinoda et al., 2009). In summary, four cornerstone states, E1–E1P–E2P–E2, provide alternating access and affinity for the transported ions (and counterions, if present). Inward facing (e.g. cytosolic) E1 and outward facing (e.g. extracellular) E2P conformations are coupled to ATP-dependent phosphorylation (yielding ion-occluded E1P) and dephosphorylation (to occluded E2) of an invariant catalytical aspartate, respectively.

P_IB_-ATPases are subdivided into groups based on conserved sequence motifs and the selectivity towards transported transition metal ions (Smith et al., 2014; Argüello, 2003; Zielazinski et al., 2012; Zhitnitsky and Lewinson, 2014). Whereas Cu^+^- and Zn^2+^-transporting P_IB-1_ and P_IB-2_ ATPases are relatively well characterized, little is known regarding the P_IB-4_ proteins, which comprise some of the simplest and shortest proteins within the entire P-type ATPase superfamily (Smith et al., 2014). They are present in plants, archaea, and prokaryotes, and have been assigned a role as virulence factors in pathogens, as for example the P_IB-4_-ATPase MtCtpD is required for tuberculosis infections (Sassetti and Rubin, 2003; Joshi et al., 2006), and therefore represent attractive targets for novel antibiotics.

The P_IB-4_-ATPases are classically referred to as cobalt transporters. However, the metal specificity of the P_IB-4_-ATPases remains elusive as some members have a confirmed cobalt specificity, while others seemingly have broader or altered ion transport profiles, also transporting ions such as Zn^2+^, Ni^2+^, Cu^+^, and even Ca^2+^ (Zielazinski et al., 2012; Raimunda et al., 2012; Moreno et al., 2008; Scherer and Nies, 2009; Seigneurin-Berny et al., 2006). Thus, the P_IB-4_-ATPases appear to have the widest scope of transported ions of the P_IB_-ATPases, and it is possible that further subclassification principles and sequence motifs will be identified. Due to the broad ion transport range, they have been proposed to serve as multifunctional emergency pumps that can be exploited under extreme environmental stress to maintain heavy-metal homeostasis (Smith et al., 2017).

Hitherto, the available high-resolution structural information of full-length P_IB_-ATPases is limited to two structures each of ion-free conformations of the Cu^+^-transporting P_IB-1_-ATPase from *Legionella pneumophila* (LpCopA) (Gourdon et al., 2011b; Wang et al., 2014), and the Zn^2+^-transporting P_IB-2_-ATPase from *Shigella sonnei* (SsZntA) (Wang et al., 2014). Thus, the principal architecture of the P_IB-4_-ATPases remains debated, as sequence analyses have proposed different topologies for the N-terminus: with or without (1) the so-called HMBDs, and (2) the first two transmembrane helices, MA and MB (Smith et al., 2014; Andersson et al., 2014; Rosenzweig and Argüello, 2012; Drees et al., 2015), which both are present in other P_IB_-ATPases (Figure 1—figure supplement 1a). These represent structural features that have been suggested to be important for ion-uptake and/or regulation in other P_IB_-ATPases (Gourdon et al., 2011b; Wang et al., 2014; González-Guerrero and Argüello, 2008; Mattle et al., 2013), raising questions if similar levels of protein control are absent or replaced in the P_IB-4_ group. In addition, despite a shared overall architecture, the P_IB-1_ and P_IB-2_ structures suggested significantly different types of entry and exit pathways, hinting at unique translocation mechanisms for each P_IB_ group (Sitsel et al., 2015). However, it remains unknown if similar molecular adaptions have taken place in P_IB-4_-ATPases to handle the unique array of cargos. To address these fundamental questions, we determined structures of a P_IB-4_-ATPase in different states and validated our findings using in vitro functional characterization.

## Results and discussion

### Metal specificity

We employed the established P_IB-4_ model sCoaT (UniProt ID A3T2G5) to shed further light on the structure and mechanism of the entire P_IB-4_-class. As the metal ion specificity of the P_IB-4_-ATPases is known to be wide, the ATPase activity was assessed in vitro in lipid–detergent solution using the so-called Baginski assay, in the presence of a range of different heavy metals. The protein exhibited clear Zn^2+^- and Cd^2+^-dependent ATPase activity, while Co^2+^ only stimulated ATP hydrolysis at high ion concentrations (Figure 2—figure supplement 1). This is in partial agreement with the ion range profile previously reported for sCoaT, as higher Co^2+^ sensitivity has been detected using different functional assays and different experimental conditions (Zielazinski et al., 2012; Figure 2—figure supplement 1).

The fact that the *K*_M_ value for the Co^2+^-dependent sCoaT activity reported previously is lower than measured in this study is unexpected (Figure 2—figure supplement 1b; Zielazinski et al., 2012). We therefore assessed if this observation relates to lower available concentration of Co^2+^ consequent to chelation by buffer solution components, or if this metal interferes with the colour development in the ATPase assays determining P_i_ concentrations (Figure 2—figure supplement 1c). However, Co^2+^ and Zn^2+^ display similar Baginski colour development as determined by calibration with separate standard curves. Moreover, neither exclusion of azide and molybdate to avoid possible Co^2+^ binding of these compounds, nor supplementation of the reducing agent TCEP (to avoid possible oxidation of Co^2+^ from molecular oxygen) has a significant effect on turnover. We also investigated if the type of assay may affect the outcome (Figure 2—figure supplement 1c). However, employment of the alternative Malachite Green Phosphate Assay essentially reproduced the relative activity in the presence of Zn^2+^ and Co^2+^, respectively (Lanzetta et al., 1979). sCoaT is purified in a buffer containing 5 mM β-mercaptoethanol, and even following dilution into the assay buffer the concentration is still approximately 100 µM, and as thiols can act as ligands for Co^2+^ it may explain part of the differences in the *K*_M_ values. However, this still does not explain why Zn^2+^- and Cd^2+^-dependent ATPase activity has not been observed for sCoaT in the previously study (Smith et al., 2017), although other P_IB-4_-members have been associated with Zn^2+^ activity. While not detected, the reported *K*_M_ and *V*_max_ may nevertheless be influenced by numerous environmental factors not tested for here, such as lipids, detergents, presence/absence/location of metal-binding his-tags, or other settings.

Despite that higher sensitivity has been measured for Zn^2+^ compared to Co^2+^, it cannot be excluded that Co^2+^, rather than Zn^2+^, is the preferred cargo in vivo as the relative intracellular availability of Co^2+^ is more than three orders of magnitude higher than that of Zn^2+^ in certain bacterial cells (Osman et al., 2019).

### Structure determination

We determined structures of sCoaT in metal-free conditions supplemented with two different phosphate analogues, BeF_3_^−^ and AlF_4_^−^, respectively, which previously have been exploited to stabilize E2 reaction intermediates of the transport cycle of P_IB_-ATPases (Wang et al., 2014; Gourdon et al., 2011b; Andersson et al., 2014). The structures were determined at 3.1 and 3.2 Å resolution, using molecular replacement (MR) as phasing method and SsZntA as search model, and the final models yielded *R*/*R*_free_ of 24.4/26.8 and 21.8/25.5 (Table 1). The two crystal forms were obtained using the HiLiDe method (crystallization in the presence of high concentrations of detergent and lipids) (Gourdon et al., 2011a). Surprisingly however, the crystal packing for both structures reveal only minor contacts between adjacent membrane-spanning regions, which are critical for the crystals obtained of most other P-type ATPase proteins (Wang et al., 2014; Gourdon et al., 2011b; Sørensen et al., 2006; Figure 1—figure supplement 2). Hence, some crystal forming interactions likely take place through lipid–detergent molecules. To our knowledge, this is the first time that type I crystals with unrestrained transmembrane domains are reported, but a consequence is that peripheral parts of the membrane domain are less well resolved (Figure 1—figure supplement 3). While this caused difficulty in modelling some transmembrane (TM) helices, satisfying solutions were found with the aid of the software ISOLDE (Croll, 2018) due to its use of AMBER forcefield which helped to maintain physical sensibility in the lowest resolution regions. In addition, root means square deviation, secondary structure as well as centre-of-mass of the transmembrane helices, only showed minor variation over time in MD simulations, indicative of a stable structure (Figure 1—figure supplements 4 and 5). The TM helices also showed lowered backbone root mean square fluctuation compared to more dynamic regions, such as the soluble domains and loop regions (Figure 1—figure supplement 4b).

**Table 1.** Data collection and refinement statistics. Statistics for the highest resolution shell are shown in parentheses.

	E2-BeF_3_^−^	E2-AlF_4_^−^
**Data collection**		
Wavelength (Å)	1.0	1.0
Space group	P 21 21 2	P 21 21 2
Cell dimensions		
*a*, *b*, *c* (Å)	89.0 94.5 128.8	89.6 93.7 128.3
a, b, *g* (°)	90 90 90	90 90 90
Resolution (Å)	47.3–3.1(3.22–3.11)	45.6–3.3(3.37–3.25)
*R*_merge_ (%)	11.4 (276.3)	15.5 (246)
*I* / σ*I*	17.8 (1.12)	8.5 (0.98)
CC_1/2_	1 (0.475)	0.99 (0.37)
Completeness (%)	97.3 (99.8)	99.2 (99.9)
Redundancy	13.3 (13.8)	6.1 (6.6)
		
**Refinement**		
Resolution (Å)	47.3–3.1(3.22–3.11)	45.6–3.3(3.37–3.25)
No. reflections	19,643 (1963)	17,466 (1714)
*R*_work_ / *R*_free_ (%)	24.4/26.8	21.8/25.5
*No. of atoms*		
Protein	4,695	4,695
Ligand/ion	5	6
Water	10	0
*Average B-factors*		
Protein	135.91	152.54
Ligand/ion	84.15	86.47
Solvent	79.62	
*R.m.s. deviations*		
Bond lengths (Å)	0.004	0.003
Bond angles (°)	0.77	0.83
*Ramachandran statistics*		
Favoured (%)	97.8	96.9
Allowed (%)	2.2	3.1
Outliers (%)ClashscoreMolProbity score	0.01.050.85	0.07.891.62

### Overall structure, without classical HMBD

**Figure 1.**
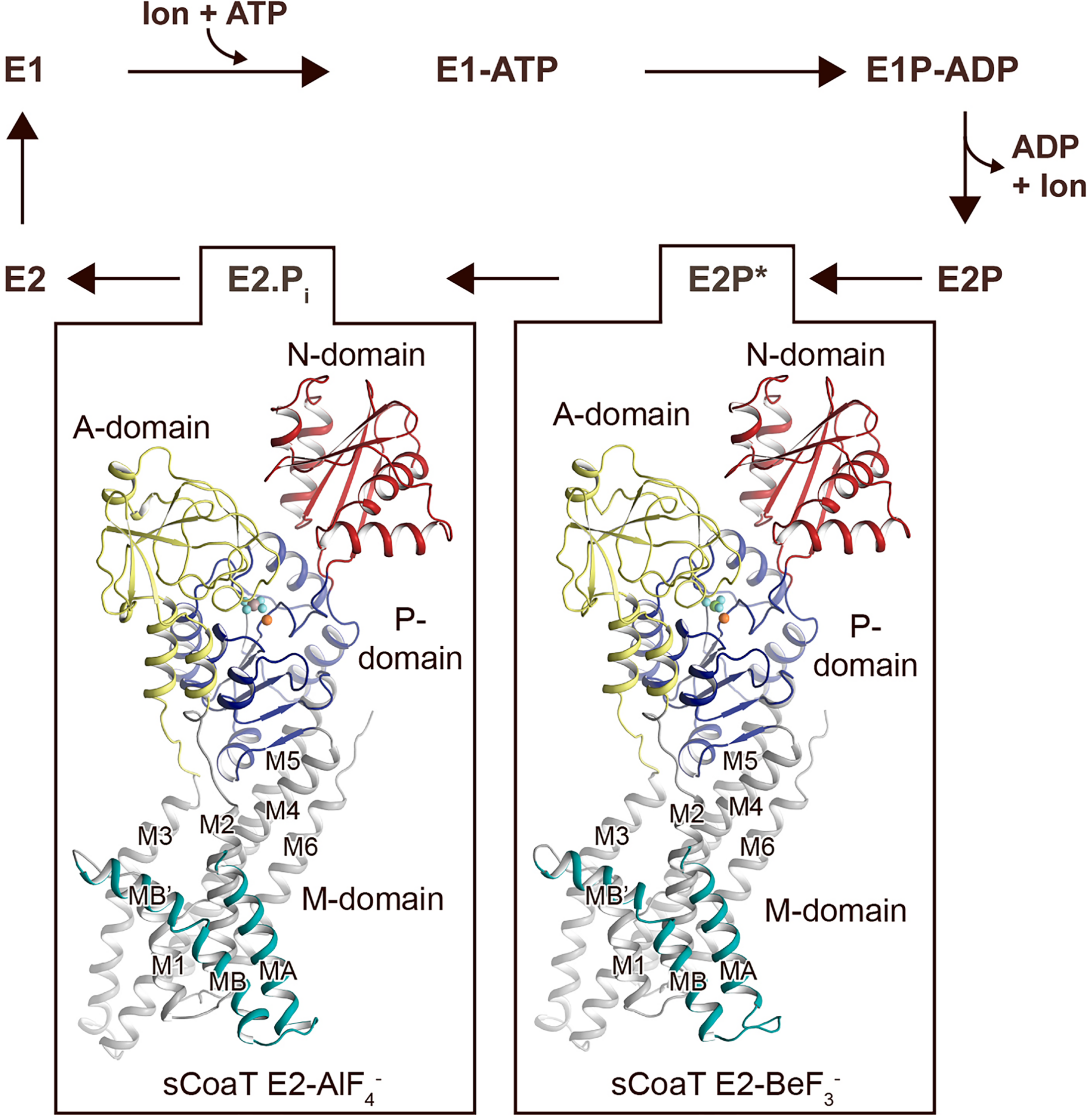
Overall architecture and reaction cycle. The sCoaT structures reveal that P_IB-4_-ATPases comprise soluble A-, P-, and N-domains, shown in yellow, blue, and red, respectively, as well as a transmembrane domain with eight helices: MA and MB, in cyan, and M1–M6, in grey, and that the P_IB-4_-topology lacks classical so-called heavy-metal-binding domain. The transport mechanism of P-type ATPases depends on ATP-dependent phosphorylation and auto-dephosphorylation, and includes four principal conformations, E1, E1P, E2P, and E2, where P denotes phosphorylation. The determined structures are trapped in two transition states following ion release – an occluded late E2P (E2P*) and an occluded transition state of dephosphorylation, E2.P_i_.

Examination of the structures reveals that the P_IB-4_-ATPase architecture is reminiscent to that of other P-type ATPases, with three cytosolic domains, A (actuator), N (nucleotide-binding), and P (phosphorylation), as well as a membrane-spanning M-domain (Figure 1a). Furthermore, the core of the soluble portions, including the nucleotide-binding pocket and catalytic phosphorylation site at D369, are well conserved.

**Figure 1—figure supplement 1.**
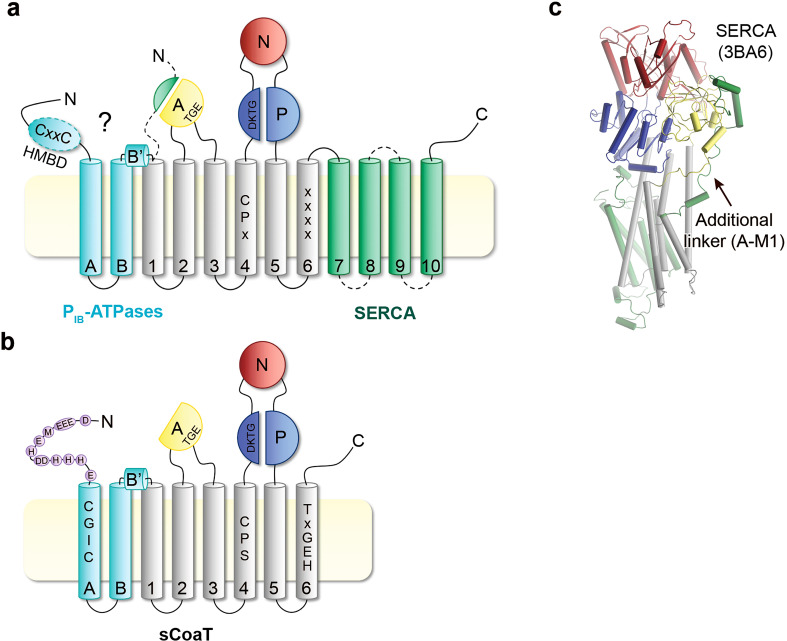
Topology comparison. Topological differences between P_IB_-ATPases and classical P-type ATPases such as sCoaT and SERCA, respectively. (**a**) Schematic topology of P-type ATPases showing features unique to P_IB_-ATPases (the so-called heavy-metal-binding domain, HMBD, and transmembrane helices MA–MB in cyan) and SERCA (an extended A-domain, an additional A-domain linker, and M7–M10 transmembrane helices in green). Location of key residues in the M-domain for P_IB_-ATPases are highlighted. (**b**) The structure of SERCA (PDB ID 3BA6), coloured as the schematic topology highlighting the additional linker to the A-domain. (**c**) Topology of the P_IB-4_-ATPase sCoaT. The present work discloses the presence of helices MA, MB, MB’, and that the core of P_IB-4_-ATPases is devoid of classical HMBD, representing a previously elusive matter. The cysteine pair (CGIC in the sequence) in the N-terminus of sCoaT is rather positioned in MA. The N-terminus of sCoaT is rich in methionine, cysteine, histidine, aspartate, and glutamate residues (shown as purple circles), amino acids that can bind metal.

**Figure 1—figure supplement 2.**
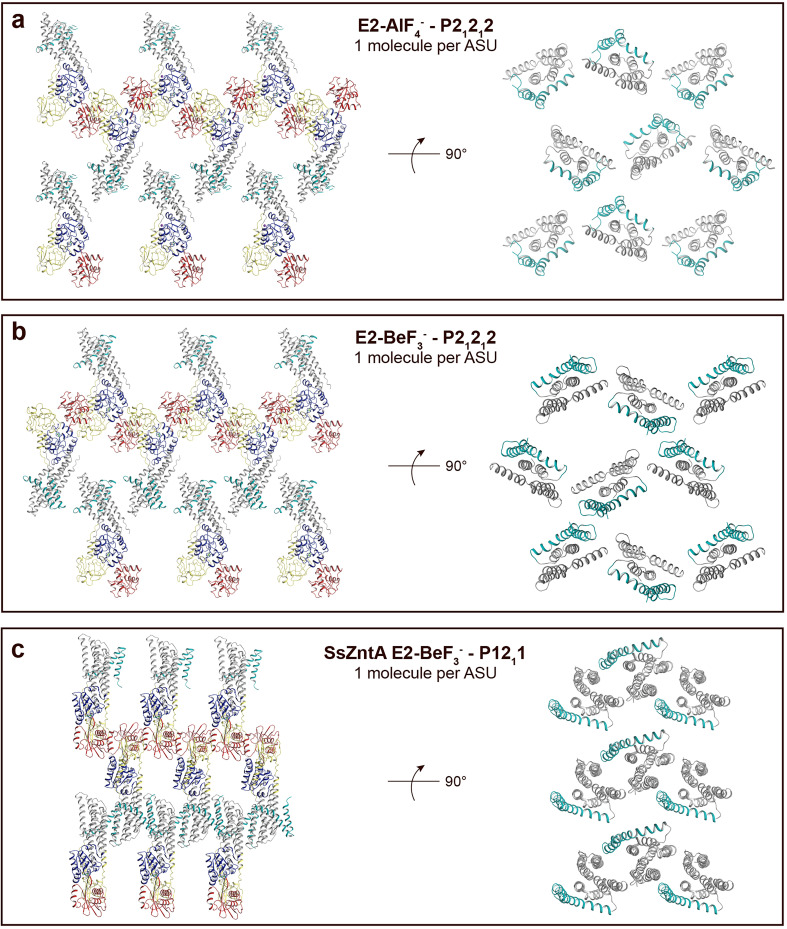
Crystal packing of sCoaT E2-AlF_4_^−^ compared to the E2-BeF_3_^−^ crystal form of ZntA from *Shigella sonnei* (SsZntA, PDB ID: 4UMV). The domains are coloured as in Figure 1b. (**a**) sCoaT E2-AlF_4_^−^ (P2_1_2_1_2, with one molecule per asymmetric unit). Left: view of the membrane layer. Right: 90° rotation view (compared to panel to the left) showing only the transmembrane domains. Equivalent views of the sCoaT E2-BeF_3_^−^ (P2_1_2_1_2) (**b**) and the SsZntA E2-BeF_3_^−^ (P12_1_1) (**c**) crystal forms. Note the loose packing of the sCoaT crystals compared to that of SsZntA E2-BeF_3_^−^.

**Figure 1—figure supplement 3.**
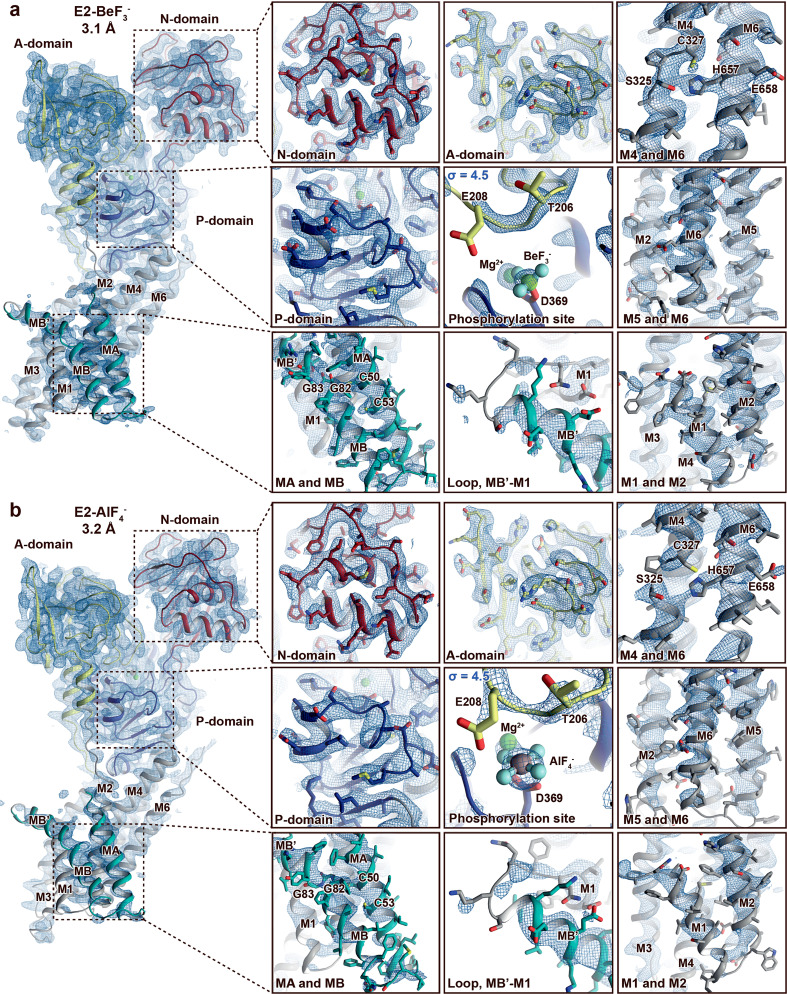
Electron density quality. Final, sharpened, 2Fo-Fc electron density at *σ* = 1.0 (blue mesh) if not otherwise stated. The overall resolution is indicated and the structures are coloured as in Figure 1. (**a**) E2-BeF_3_^−^ and (**b**) E2-AlF_4_^−^. The quality of the maps differs between structures and domains. The A-, P-, and N-domains are well resolved in both structures. The M-domain is in general less well resolved than the soluble domains, and the domain is somewhat more well resolved in the E2-BeF_3_^−^ structure than in E2-AlF_4_^−^ structure. Nevertheless, it is still clear that MA and MB are present and that C50 and C53 in the N-terminus are membrane embedded and not part of a heavy-metal-binding domain (HMBD). This is relevant, as CXXC is otherwise a pattern typically linked to a solvent-exposed metal-binding site in HMBDs of other P_IB_ groups.

**Figure 1—figure supplement 4.**
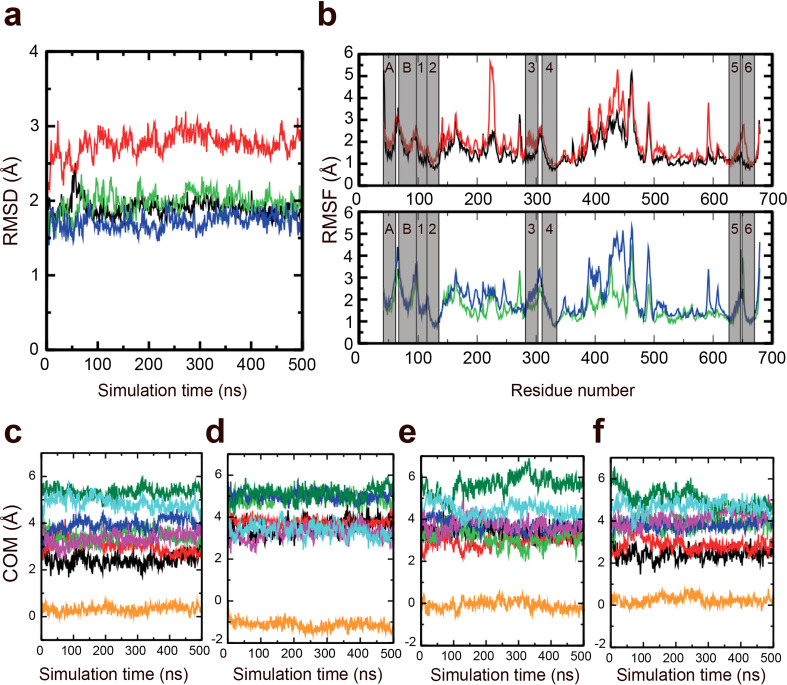
Stability of the M-domain. Molecular dynamics (MD) simulations were performed to assess the stability of the M-domain. (**a**) Root mean square deviation of the M-domain in AlF_4_^−^ (black), AlF_4_^−^-repeat (red), BeF_3_^−^ (green), and BeF_3_^−^-repeat (blue) simulations. (**b**) RMSF of the M-domain in the AlF_4_^−^ and AlF_4_^−^-repeat simulations (upper) and BeF_3_^−^ and BeF_3_^−^-repeat simulations (lower) across the C_α_ atoms. The transmembrane (TM) helices region are marked in transparent grey. Evolution of the centre-of-mass of TM helices in the (**c**) AlF_4_^−^, (**d**) AlF_4_^−^-repeat, (**e**) BeF_3_^−^, and (**f**) BeF_3_-repeat simulations. The TM helices are shown in different colours: MA (black), MB (red), M1 (green), M2 (blue), M3 (magenta), M4 (orange), M5 (dark green), and M6 (cyan).

**Figure 1—figure supplement 5.**
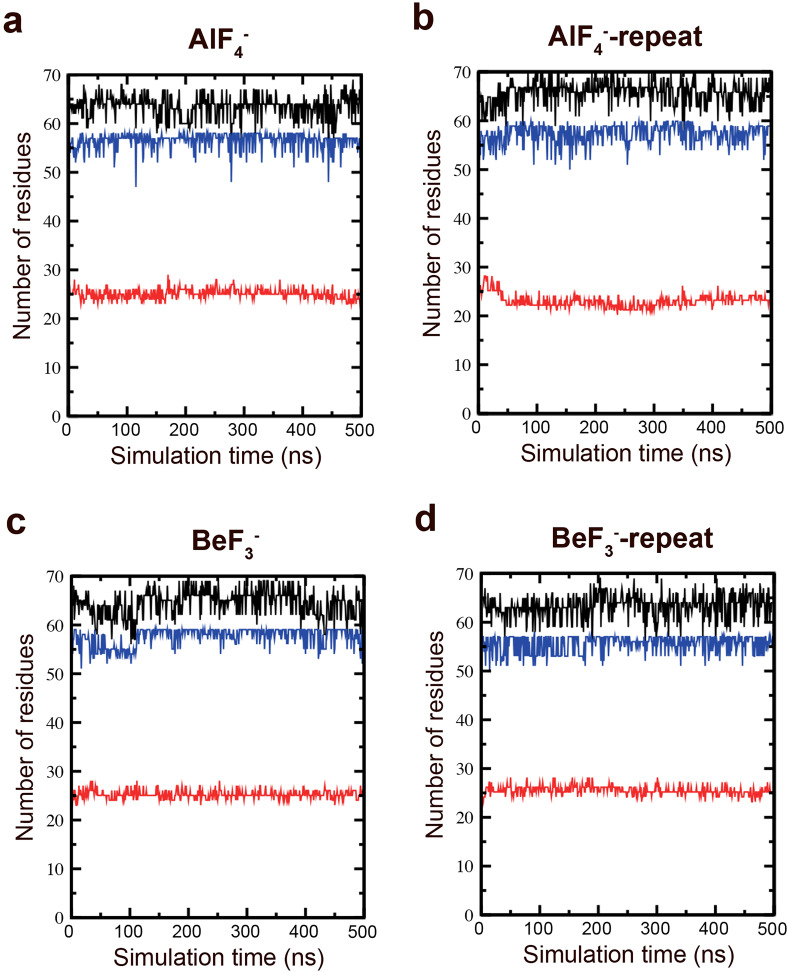
Secondary structure stability of the M-domain. MD simulations were performed to assess the secondary structure stability of the M-domain. Total structure (black), helix (blue), and coil (red) secondary structural elements in the (**a**) AlF_4_^−^, (**b**) AlF_4_^−^-repeat, (**c**) BeF_3_^−^, and (**d**) BeF_3_^−^-repeat simulations.

The topology of P_IB-4_-ATPases has been a conundrum as sequence analyses have proposed different arrangements, with variable number of transmembrane segments and different sizes of the N-termini (Smith et al., 2014; Gourdon et al., 2011b; Wang et al., 2014; Rosenzweig and Argüello, 2012; Drees et al., 2015; Andersson et al., 2014). However, our data unambiguously demonstrate that P_IB-4_-ATPases possess eight transmembrane helices, MA and MB followed by M1–M6. As previously observed for P_IB-1_- and P_IB-2_-ATPases, MB is kinked by a conserved Gly–Gly motif (G82 and G83), forming an amphipathic ‘platform’, MB’, immediately prior to M1, see further below (Figure 1—figure supplement 3).

**Figure 2.**
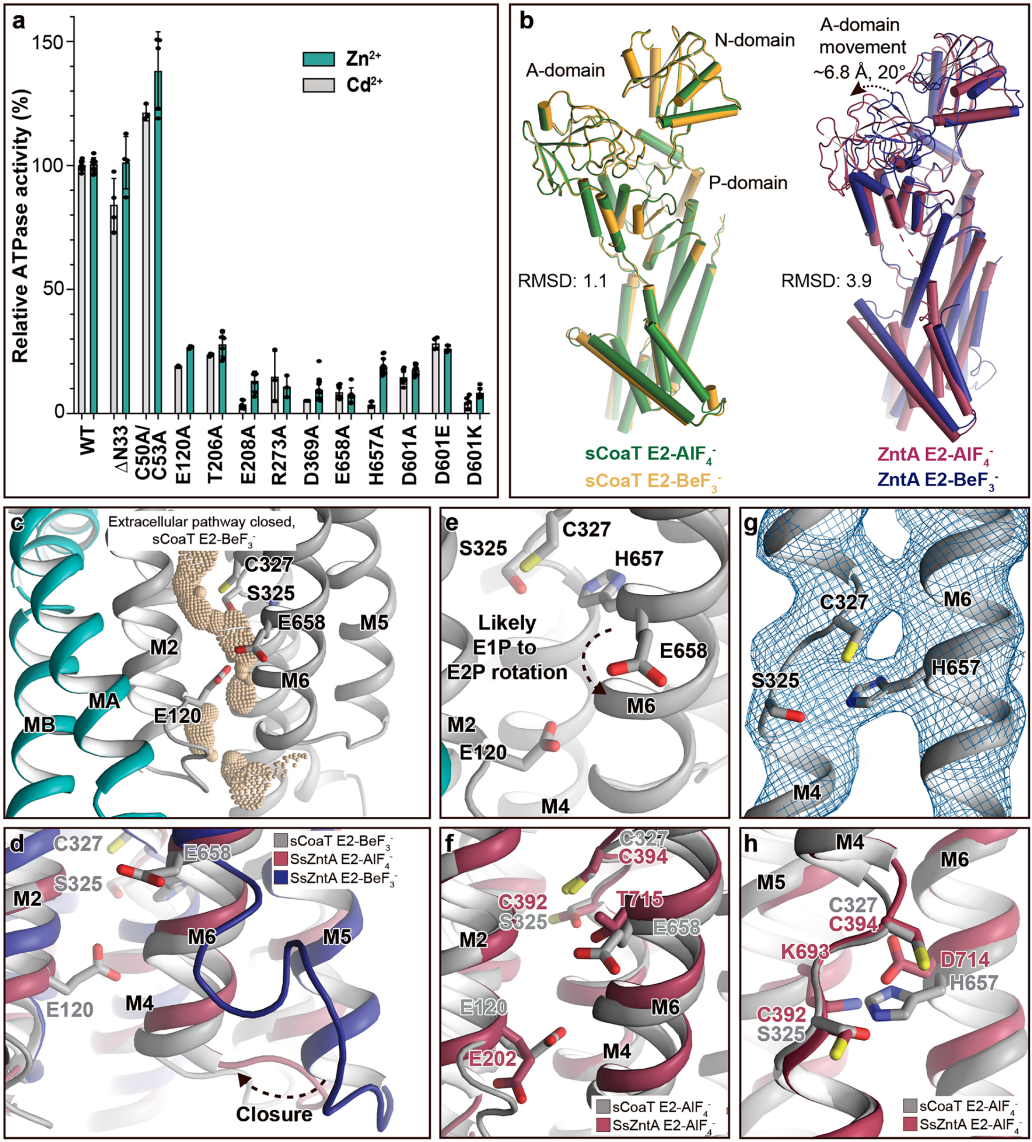
Mechanistic insight into the function of P_IB-4_-ATPases. (**a**) Functional ATPase assay in lipid–detergent solution with targeted residues in sequential order. The wild-type (WT)-specific activity using the employed experimental conditions in the presence of 50 μM metal is 1.00 ± 0.01 μmol mg^−1^ min^−1^ with Zn^2+^ and 2.80 ± 0.06 μmol mg^−1^ min^−1^ with Cd^2+^, comparable to the activity previously measured for P_IB-4_-ATPases. For biological averages and SD, see Figure 2—figure supplement 1e. (**b**) Comparisons of E2-AlF_4_^−^ and E2-BeF_3_^−^ structures of sCoaT and the equivalent of SsZntA (PDB ID of SsZntA structures: 4UMV and 4UMW). All superimpositions were performed based on the P-domain, and the RMSD values for the overall structures are indicated. (**c**) Identified cavity (wheat) in the E2-BeF_3_^−^ structure using the software HOLE. The E2-BeF_3_^−^ and the E2-AlF_4_^−^ (not shown) structures are occluded, lacking continuous connection between the ion-binding site to the outward environment. (**d**) The conformational changes that likely allow for closure of the release pathway, as illustrated from the E2-BeF_3_^−^ structure of SsZntA to the E2-AlF_4_^−^ structures of sCoaT or SsZntA. (**e–h**) Close views of ion-binding and -release residues in the M-domain of sCoaT and SsZntA. (**e**) The orientation of E658 is incompatible with high-affinity binding, and is likely contributing to ion release. (**f**) Release likely takes place via E658 and E120. (**g**) The sandwiched position between S325 and C327 of H657, including the final 2Fo-Fc electron density (blue). (**h**) The position of H657 in sCoaT overlaps with the one of K693 in SsZntA, and both likely serve as in-built counterions.

Are then HMBDs present in P_IB-4_-ATPases as in the other P_IB_ subclasses? As only the first 47 residues remain unmodelled in the final structures (Table 1), it is clear that many P_IB-4_-ATPases including sCoaT are lacking a classical HMBD ferredoxin-like fold (typically 70 residues long). In agreement with this observation, the cysteine pair (CGIC in the sequence) in the N-terminus of sCoaT is rather positioned in MA, facing M1 (Figure 1—figure supplements 1 and 3), in contrast to the surface-exposed, metal-binding CXXC hallmark-motif detected in classical HMBDs. Functional analysis of mutant forms lacking these cysteines in vitro also support that they are unimportant for function (Figure 2a). We note that there are P_IB-4_-ATPases with extended N-termini that, in contrast to sCoaT, may harbour HMBDs (Smith et al., 2014). Conversely, the sCoaT N-terminus is rich in metal-binding methionine, cysteine, histidine, aspartate, and glutamate residues, and this feature is conserved among P_IB-4_-ATPases (Figure 2—figure supplement 2). We therefore explored the role of this N-terminal tail through assessment of an sCoaT form lacking the first 33 residues. However, in vitro characterization suggests only minor differences compared to wild-type, indicating that the residues upstream of MA are not essential for catalytic activity (Figure 2a). Aggregated, this hints at that no classical HMBD is present, and hence that this level of regulation is absent in many P_IB-4_-ATPases, although it cannot be excluded that the N-termini are important in vivo.

**Figure 2—figure supplement 1.**
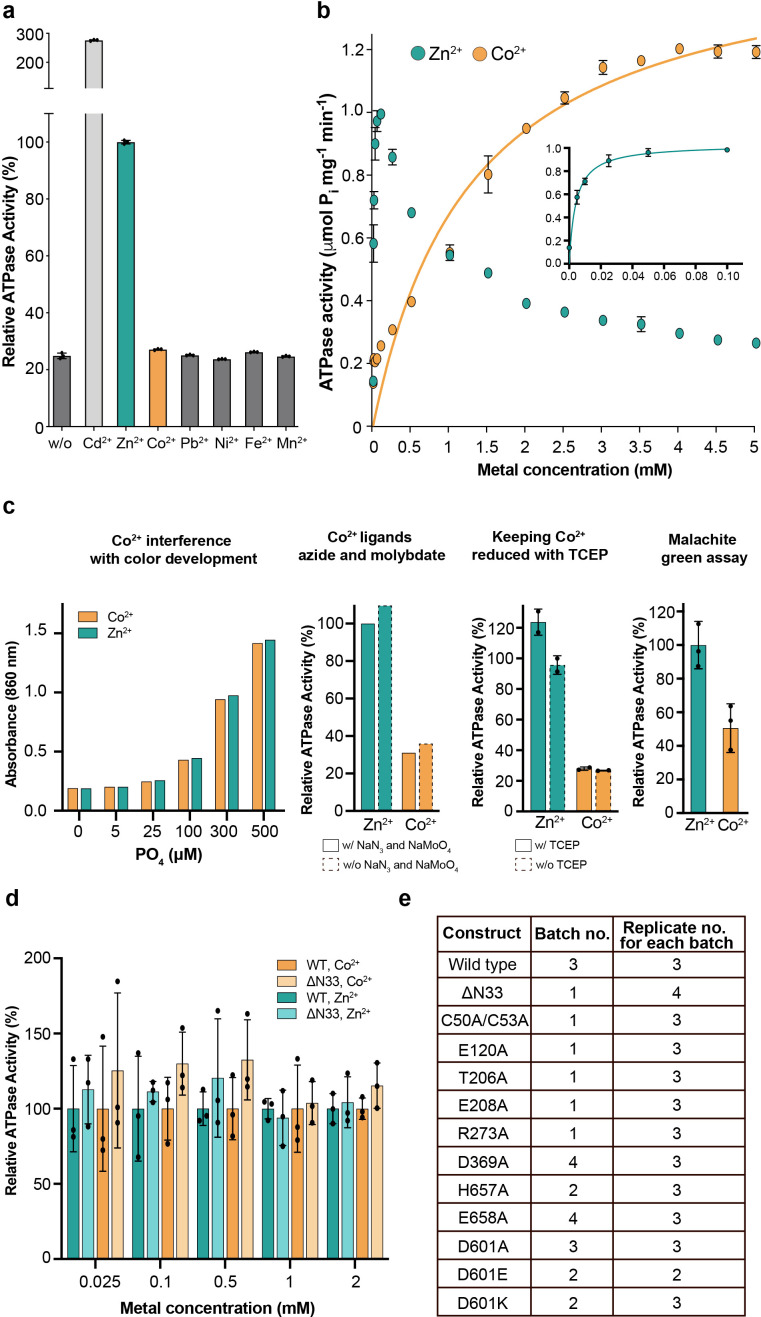
Metal selectivity screening and reproducibility. (**a**) Screen of different transition metals, tested at 50 μM each. There is clear metal-dependent ATPase activity with Zn^2+^ (1.0 ± 0.01 µmol mg^−1^ min^−1^) and Cd^2+^ (2.8 ± 0.05 µmol mg^−1^ min^−1^), comparable to the activity previously measured for P_IB-4_-ATPases and also to Zn^2+^-dependent activity of SsZntA (0.59 ± 0.02 μmol mg^−1^ min^−1^) Wang et al., 2014. In contrast, only low ATPase activity (about 5% of the wild-type, corrected for the background observed with no metal added) was detected with Co^2+^ for sCoaT. (**b**) Titration of zinc and cobalt. Co^2+^-induced ATPase activity predominates above 1 mM, while at lower concentrations Zn^2+^ stimulates activity at a faster rate. The data yield *K*_M_ values of 1.3 mM and 4.1 µM for Co^2+^ and Zn^2+^, respectively. The corresponding for the SsZntA related pump EcZntA is 10 µM with Zn^2+^ (Mitra and Sharma, 2001). (**c**) Screening of assay conditions and assay type. (**d**) The effect of the N-terminal tail on the ion specificity. Relative activity in the presence of Co^2+^ and Zn^2+^ (100% is equal to the activity measured for wild-type at every measured metal type and concentration), respectively, at five different metal concentrations, suggesting that the tail is no major determinant for metal specificity. (**e**) Biological and technical replicates exploited to generate the error bars in Figure 2a.

**Figure 2—figure supplement 2.**
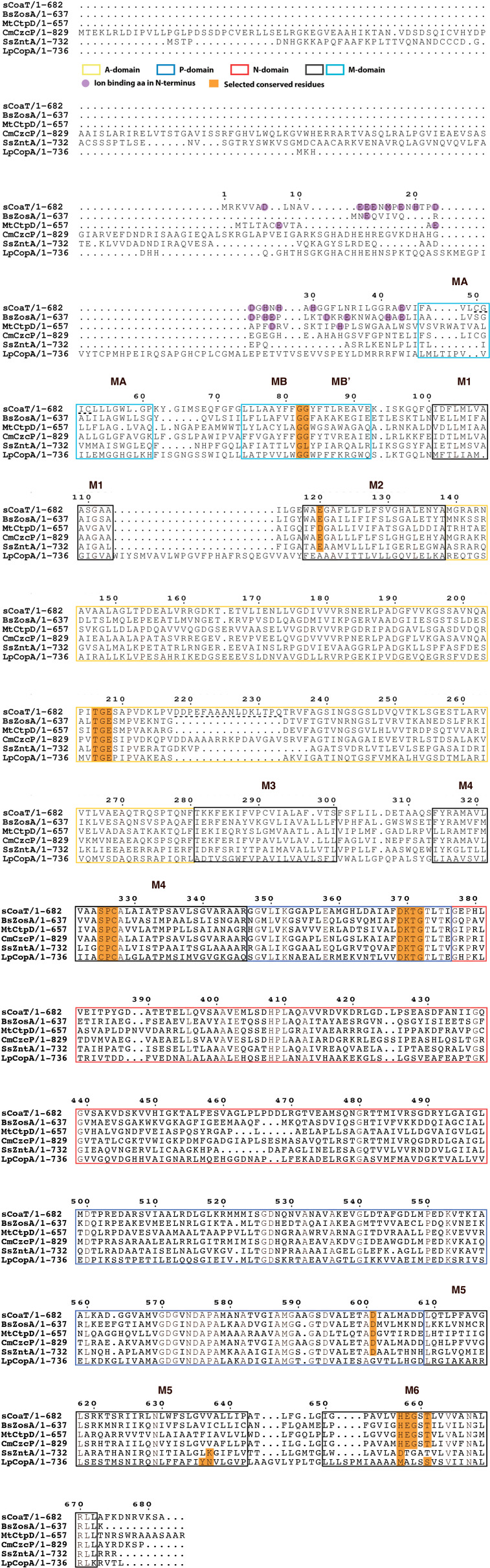
Sequence alignment of selected P_IB_-ATPases. Sequence alignment of four P_IB-4_-ATPases, sCoaT from *Sulfitobacter* sp. NAS-14.1 CmCzcP from *Cupriavidus metallidurans*, BsZoa from *Bacillus subtilis* and MtCtpD from *Mycobacterium tuberculosis*. The P_IB-1_-ATPase LpCopA from *Legionella pneumophila* and the P_IB-2_-ATPase SsZntA from *Shigella sonnei* are also included for comparison.

**Figure 2—figure supplement 3.**
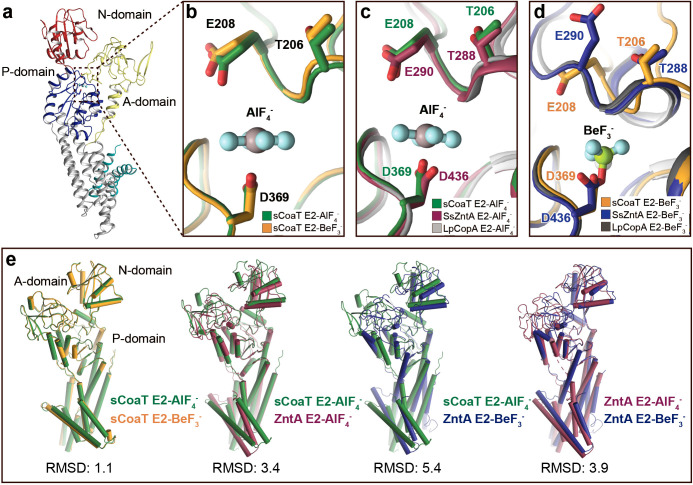
Comparison of E2 states overall and close views of the phosphorylation site. The TGE loop in the E2-BeF_3_^−^-stabilized sCoaT (E2P*) is preorganized for dephosphorylation, which is not the case for SsZntA and LpCopA. (**a**) The overall E2P* structure of sCoaT showing the region of focus in panels b–d. (**b**), Comparison of the TGE loop in the two sCoaT structures, with only minor differences. (**c**) Comparison of sCoaT E2.P_i_ with the equivalent structures of SsZntA and LpCopA (PDB ID: 4UMW and 4BYG). (**d**) Comparison of sCoaT E2P* with the equivalent structures of SsZntA and LpCopA (PDB ID: 4UMV and 4BBJ). (**e**) Comparisons of E2-AlF_4_^−^ and E2-BeF_3_^−^ structures of sCoaT and SsZntA (PDB ID of SsZntA structures: 4UMV and 4UMW). All superimpositions were performed based on the P-domain, and the RMSD values based on the overall structure are listed below the structural alignments. Alignment of the E2-BeF_3_^−^ and E2-AlF_4_^−^ structures of sCoaT demonstrates that they are very similar (RMSD = 1.1), and comparison to the equivalent structures of SsZntA support the conclusion from (**a–d**) that both structures have been captured in occluded E2.P_i_ transition states.

**Figure 2—figure supplement 4.**
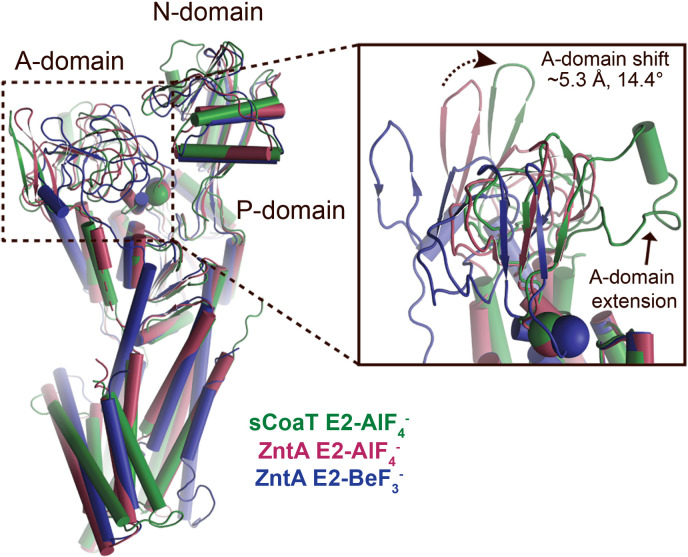
A-domain differences. Superimposition of the E2-AlF_4_^−^ structures of sCoaT (determined here, shown in green) and SsZntA (PDB ID 4UMW, purple) and the E2-BeF_3_^−^ structure of SsZntA (PDB ID 4UMV, blue). The overall structures are shown to the left. The inset represents a close-view of the A-domain, showing that the sCoaT structure is more alike the SsZntA E2-AlF_4_^−^ structure. The peripheral part of the A-domain in sCoaT is shifted closer to the P-domain, whereas the area around the conserved TGE motif (the Glu of the TGE motif is visualized as a sphere) superposes well with SsZntA. Like SERCA, the A-domain of sCoaT possesses a surface-exposed extension which is however not present in P_IB-1_- and P_IB-2_-ATPases.

Interestingly, it has been shown that the in vivo transport specificity of the sCoaT homolog from *Synechocystis PCC 6803* (CoaT) can be switched from Co^2+^ to Zn^2+^ by exchanging the N-terminal region to that of the Zn^2+^ transporting P_IB-2_ ATPase ZiaA from same organism (Borrelly et al., 2004). This demonstrates that P_IB-4_-ATPases not only in vitro (our data), but also in vivo are able to transport Zn^2+^, if the M-domain gain access to the metal. One possible explanation for the change of specificity for the CoaT chimeric construct is that the N-terminal peptide tail, as also suggested for ATP7B (Yu et al., 2017), prevents ATP hydrolysis through binding to the soluble domains, and this inhibition is then released upon binding of the cognate metal to the N-terminal and/or HMBD. However, it is also possible that the role of the N-terminal region of P_IB-4_ proteins is to impair Zn^2+^ acquisition, an ability that is lost when exchanged with the N-terminal part of ZiaA. Preliminary assessment of the metal specificity influence of the N-terminal tail of sCoaT suggests it has little or no effect on distinguishing between Co^2+^ and Zn^2+^ in vitro (Figure 2—figure supplement 1d). From this it is clear that further studies are needed to shed light on the function of the N-terminal region in P_IB_-ATPases, also in P_IB-4_-ATPases.

Associated, this raises questions also on the role of the above-mentioned MB’ platform, which has been proposed to serve as an interaction site for HMBDs in P_IB-1_- and P_IB-2_-ATPases, and for the Cu^+^-ATPases as a docking site for metal delivering chaperones (Gourdon et al., 2011b; Wang et al., 2014; González-Guerrero and Argüello, 2008; Morin et al., 2009). As there are no known zinc/cadmium chaperones for P_IB-4_-ATPases, and because classical HMBDs appear to be missing in at least some proteins of the group, the MB’ function may need to be revisited. Alternatively, the N-terminus may have merely been maintained through evolution without conferring functional benefits or disadvantages.

### Structures in a transition state of dephosphorylation

The classical view of P-type ATPases is that the E2P state is outward open and that the following transition state of dephosphorylation, E2.P_i_, is occluded, and that these conformations can be stabilized using the phosphate analogues employed here for structure determination, BeF_3_^−^ and AlF_4_^−^, respectively. Furthermore, distinct ion-release pathways have been proposed among P_IB_-ATPases (Gourdon et al., 2011b; Wang et al., 2014; Andersson et al., 2014; Mattle et al., 2015), including a narrow exit pathway lined by MA, M2, and M6 that remains open also in the E2.P_i_ state for the P_IB-1_-ATPases. In contrast, a wide opening extending from the location of the bound metal in the M-domain of ion-occluded states to the non-cytoplasmic side has been observed for the P_IB-2_-ATPases, and this group becomes reoccluded with the E2P to E2.P_i_ shift.

Surprisingly however, analysis of the two obtained structures suggests that the anticipated significant domain reorientations are absent in sCoaT (Figure 2b), and the models are in contrast rather similar. The compact assembly of the soluble domains and the position of the A-domain near the P-domain, placing the conserved TGE motif responsible for dephosphorylation towards the phosphorylation site, are typically associated with commencement of dephosphorylation, indicating that the two structures are trapped in an E2.P_i_ like transition state (Figure 2—figure supplement 3a, c, e). This observation differs from the equivalent structures of the other structurally determined P_IB_-ATPases, in which the phosphorylation site of the E2P state (stabilized by BeF_3_^−^) is shielded from the TGE loop as also observed for the well-studied sarcoendoplasmic reticulum Ca^2+^-ATPase (SERCA) (Figure 2—figure supplement 3d).

Notably, analogous highly similar BeF_3_^−^- and AlF_4_-stablized structures have recently also been observed for the Ca^2+^-specific P-type ATPase from *Listeria monocytogenes* (LMCA1) (Hansen, 2020). It was proposed that LMCA1 preorganizes for dephosphorylation already in a late E2P state (E2P*, stabilized by BeF_3_^−^), in accordance with its rapid dephosphorylation. Favoured occlusion and activation of dephosphorylation directly upon ion release may thus also be the case for sCoaT, and consequently the E2-BeF_3_^−^ structure captured here may represents a late (or quasi) E2P state (E2P*).

Comparisons of the sCoaT structures to the equivalent structure of SsZntA (E2.P_i_) revealed a unique arrangement of the A-domain (Figure 2—figure supplement 4). The TGE-loop region superposes well with the corresponding area in SsZntA, but the rest of the A-domain is rotated towards the P-domain – approximately 14° and 5.3 Å (Figure 2—figure supplement 4). However, it cannot be excluded that this rotation is due to crystal contacts as the two peripheral β-sheets of the A-domain are interacting tightly with parts of a neighbouring molecule. Additionally, we noticed that the A-domain of sCoaT possesses a surface-exposed extension similar to SERCA, but this feature is not present in P_IB-1_- and P_IB-2_-ATPases and it is not a conserved property in the P_IB-4_ group either (Figure 2—figure supplements 2 and 4). Conversely, the M-domains of the two sCoaT structures are overall similar and appear outward occluded (Figure 2c), as also supported by comparisons with the equivalent structures of SsZntA, again contrasting to the situation observed in P_IB-1_- and P_IB-2_-ATPases.

### Ion release

Next, to shed light on ion release, we compared the sCoaT structures to the E2P state of SsZntA, in which the extracellular ends of M5 and M6 shifts away from the proposed metal-binding site, allowing an exit pathway to be formed (Figure 2d). Considering that P_IB-2_- and P_IB-4_-ATPases have overlapping cargo range, share overall topology and that they release ions in free form to the extracellular environment, in contrast to their P_IB-1_ counterparts, we find it likely that they employ similar exit pathways, lined primarily by M2, M4, M5, and M6 (Figure 2d; Wang et al., 2014; Andersson et al., 2014).

The high-affinity-binding site in P_IB-4_-ATPases has previously been suggested to be formed by residues from the conserved SPC- (starting from S325) and HEGxT- (from H657) motifs of M4 and M6, based on X-ray absorption spectroscopy and mutagenesis studies (Zielazinski et al., 2012; Patel et al., 2016). An outstanding remaining question is, however, how the ion is then discharged to the extracellular site? Among the resides that likely constitute the high-affinity-binding site, remarkably E658 of M6 is pointing away from the ion-binding region around the SPC motif (Figure 2e and Figure 1—figure supplement 3). We anticipate that E658 rotates away from its ion-binding configuration in the E1P to E2P transition, thereby assisting to lower the cargo affinity to permit release via the M2, M4, M5, and M6 cavity (Figure 2e). The conserved E120 of M2 (sometimes replaced with an aspartate in P_IB-4_-ATPases) is located along this exit pathway. The residue also overlays with the conserved E202 in SsZntA (Figure 2f), which has been suggested to serve as a transient metal ligand, stimulating substrate release from the CPC motif of P_IB-2_-ATPases (Wang et al., 2014). We propose a similar role for E120 in sCoaT as further supported by the decreased activity of E120A sCoaT form (Figure 2a).

### A unique internal counterion principle

Many P-type ATPases couple ion- and counter transport, and hence the reaction cycle cannot be completed without counterions. The importance of the counter transport has been demonstrated in for example Ca^2+^/H^+^- (such as SERCA), Na^+^/K^+^-, and H^+^/K^+^-ATPases (Moller et al., 2010; Faxén et al., 2011; Abe et al., 2018; Dyla et al., 2020). In contrast, the absence of counter transport has been proposed for P_IB-2_-ATPases (Wang et al., 2014), H^+^-ATPases (Pedersen et al., 2007), and P4-ATPases (Nakanishi et al., 2020), which rather exploit a built-in counterion. Specifically for the P_IB-2_-ATPases, a conserved lysine of M5 (K693 in SsZntA) serves as the counterion, through interaction with the conserved metal-binding aspartate of M6 (D714 in SsZntA) in E2 states. Similarly, P_IB-1_-ATPases are not Cu^+^/H^+^ antiporters, but a likely built-in counterion residue is not conserved in the group (Abeyrathna et al., 2020). Instead, it is possible that the requirement for counterion translocation is prevented by the narrow exit pathway, preventing backtransfer of the released ion and perhaps rendering complete occlusion unnecessary (Abeyrathna et al., 2020). For the P_IB-4_-ATPases, biochemical studies have proposed an ion-binding stoichiometry of one (Zielazinski et al., 2012; Raimunda et al., 2012; Smith et al., 2017; Patel et al., 2016), however no information is available regarding the presence or absence of counter transport.

In the E2-BeF_3_^−^ sCoaT structure, we identify a tight configuration of HEGxT-motif H657, being sandwiched between the SPC residues, distinct from the M5 lysine–M6 aspartate interaction observed in P_IB-2_-ATPases (Figure 2g, h). Despite the packing issues of the generated crystals, clear electron density is visible for H657, indicating a rigid conformation (Figure 2g). Moreover, activity measurements of an alanine substitution of H657 demonstrate that it is crucial for function (Figure 2a). In light of these findings and an earlier report suggesting that a mutation of the equivalent of H657 in MtCtpD leaves the ion affinity unaffected (Patel et al., 2016), we suggest this histidine serves as an internal counterion, similarly as for the invariant lysine in SsZntA, perhaps preventing backtransfer of released ions and for charge stabilization, however we cannot exclude that H657 is also part of the high-affinity-binding site in sCoaT.

The rigid conformation observed for H657 in the E2-BeF_3_^−^ structure is also observed in the E2-AlF_4_^−^ structure (Figure 1—figure supplement 3b). In contrast, for SsZntA the interaction between K693 and D714 is only detected in the E2.P_i_ state. Thus, the interaction pattern is consistent with the idea that sCoaT preorganizes for dephosphorylation already in the (late) E2P state, with the associated occlusion and internal counter–ion interaction taking place earlier than for SsZntA.

### A more potent A-domain modulatory site

**Figure 3.**
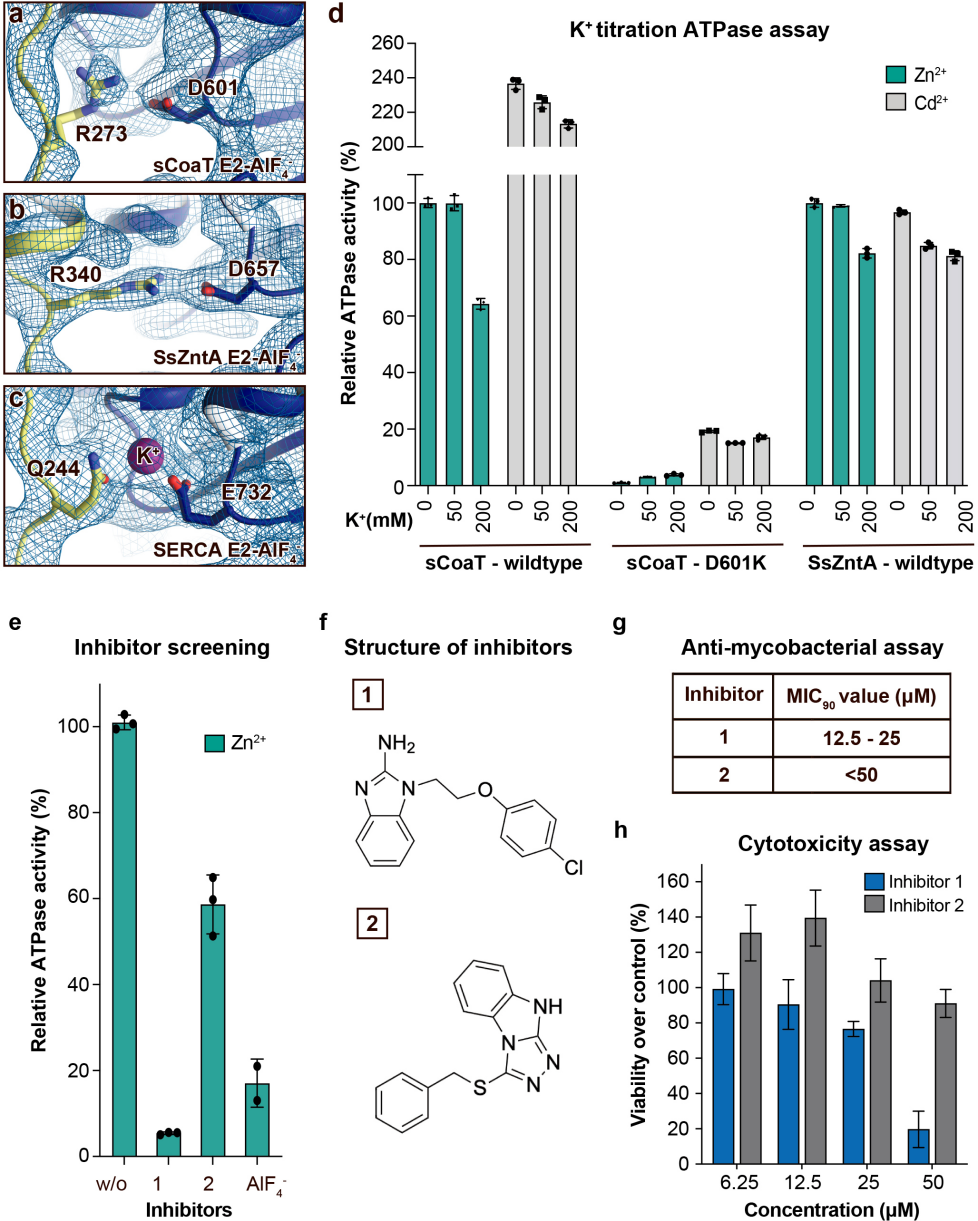
Regulation and inhibition. (**a–c**) Close views of the regulatory point-of-interaction between the A- and P-domains in the E2-AlF_4_^−^ structures of sCoaT, SsZntA, and SERCA (PDB IDs 4UMW and 1XP5) with the corresponding 2Fo-Fc electron density shown at *σ* = 1.0 (blue mesh). (**a**) sCoaT (coloured as in Figure 1) with interaction between D601 and R273. (**b**) SsZntA (shown as panel a) with interaction between D657 and R340. (**c**) SERCA (shown as in panel a) with bound K^+^ (purple) between E732 and Q244. (**d**) Functional ATPase assay in lipid–detergent solution of sCoaT (wild-type and D601K forms) as well as SsZntA (wild-type), using protein samples purified in the absence of K^+^ and Na^+^ (see Methods). The mean + SD of technical replicates is shown (*n* = 3). KCl leaves the function of sCoat and SsZntA essentially unaffected in the presence of Zn^2+^ (cyan) or Cd^2+^ (grey). The equivalent form of sCoaT D601K has previously been exploited to demonstrate K^+^ dependence in the Na,K-ATPase (Schack et al., 2008). Collectively, these data suggest that the P-/A-domain site regulation is K^+^ independent in P_IB_-ATPases, in contrast to classical P-type ATPases. (**e–h**) Evaluation of the effect on selected identified novel inhibitors on activity of protein, as well as survival of mycobacteria and primary human macrophages. (**e**) Effect of two inhibitors (300 μM) on the activity of sCoaT assessed in lipid–detergent solution in the presence of Zn^2+^. For comparison, the commonly used P-type ATPase inhibitor AlF_4_^−^ (500 μM) is included. (**f**) The structure of inhibitors 1 and 2. (**g**) The minimal inhibitory concentration to kill 90% (MIC_90_) of mycobacteria for inhibitors 1 and 2. The mean MIC_90_ value for inhibitor 1 is 18.75 µM, while for inhibitor 2 it is over 50 µM. The values are based on four separate experiments. (**h**) The cytotoxic effect of different concentrations of inhibitors 1 and 2 on primary human macrophages (ATP assay). The standard error of mean (SEM) of nine replicates is shown (*n* = 9).

A conserved K^+^ site, which cross-links between the A- and P-domains in E2 states and thereby allosterically stimulates the E2P to E2 process (Sørensen et al., 2004; Schack et al., 2008), has been suggested to be present also in P_IB_-ATPases (Sørensen et al., 2004). However, our new E2 structures and available structures of P_IB-1_- and P_IB-2_-ATPases suggest that the A-/P-domain linker is maintained without K^+^ in P_IB_-ATPases, and instead is established directly between R273/D601 in sCoaT, as also supported by potassium titration experiments monitoring sCoaT ATPase activity (Figure 3a–d). Nevertheless, the A-/P-domain point-of-interaction appears critical for P_IB_-ATPases, as functional characterization of R273A, D601A, and D601K result in a marked reduction of turnover (Figure 2a). This differs from similar mutations of classical P-type ATPases, where only minor effects are observed (Sørensen et al., 2004; Schack et al., 2008). Furthermore, substitution of D601 with glutamate suggests that even the A-/P-domain distance is critical (Figure 2a). It is possible that P_IB_-ATPases are more reliant on this particularly tight, ion-independent stabilization, as the A–M1/A-domain linker is absent, and because many other P-type ATPases also have a complementary A-/P-domain interaction (Figure 1—figure supplement 1c). Thus, our data indicate that this regulation is a general feature of many P-type ATPase classes, yet featuring unique properties for P_IB_-ATPases.

### New metal-transport blockers

P_IB-2_- and P_IB-4_-ATPases serve as virulence factors and are critical for the disease caused by many microbial pathogens, as underscored by the frequent presence of several redundant genes (Sassetti and Rubin, 2003; Joshi et al., 2006; Botella et al., 2011; Pi et al., 2016). In this light and because these P-type ATPases are missing in humans, they represent putative targets for novel antibiotics. The shared mechanistic principles identified here suggest that compounds can be identified that inhibit both P_IB_ groups, for example directed against the common release pathway, thereby increasing efficacy. Indeed, screening of a 20,000-substance library using a complementary in vitro assay, uncovers several compounds that abrogate function of sCoaT and SsZntA (Figure 3e, f, data only shown for sCoaT). Furthermore, initial tests of two of these suggest they have a potent effect against mycobacteria, which previously have been shown to be P_IB-4_ dependent for infection (Patel et al., 2016); 90% of the mycobacteria were killed at mean concentrations of 18.75 and above 50 µM, respectively, using either of these two separate molecules (Figure 3g). In contrast, investigation of cytotoxic effects on primary human macrophages at concentrations up to 25 µM demonstrated considerably less impact on cell survival for both blockers (Figure 3h). Evidently downstream in-depth studies, ranging from investigations of the target specificity, the detailed effect on human cells as well as antibiotic potency in human, are required to fully understand the value of these putative P_IB-2_- and P_IB-4_ inhibitors. Nevertheless, the substances outlined here represent promising leads for drug-discovery efforts or to aid the development of tools to manipulate heavy-metal accumulation in plants to prevent accumulation or for enrichment.

### Conclusion

**Figure 4.**
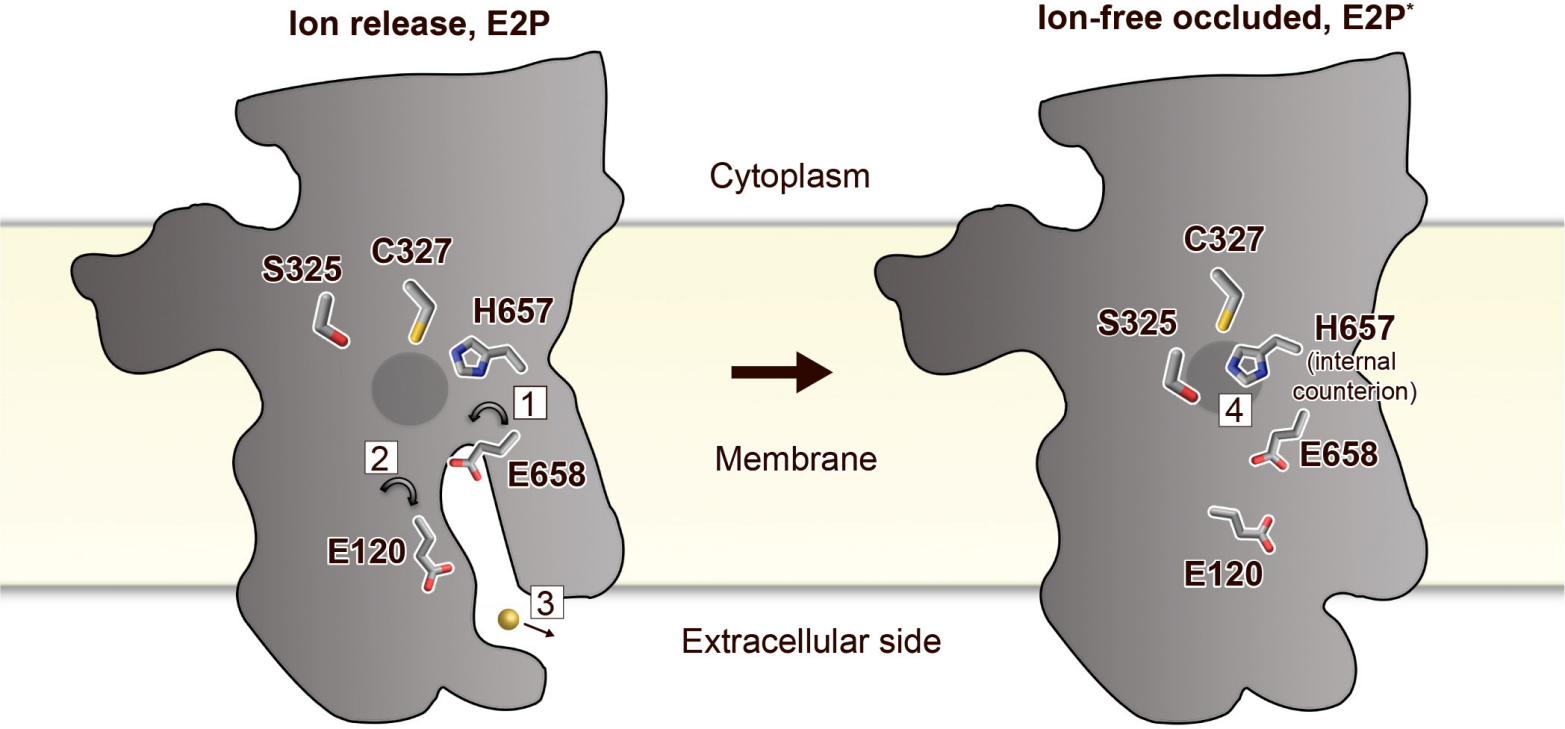
Putative ion-release and reocclusion mechanism of P_IB-4_-ATPases. Schematic model illustrating the transmembrane domain (the soluble domains have been removed for clarity) of two separate states, an E2P and an occluded E2P* conformation as the determined structure (E2-BeF_3_^−^), respectively. Zinc or cadmium release from the high-affinity-binding site in the M-domain is likely permitted through re-orientation of E658 (1) in the E1P to E2P transition, thereby lowering the affinity for the occluded ion. E120 serves as a transient linker between the high-affinity-binding site and the outward environment (2). Following ion-release (3) H657 shifts to a sandwiched position between S325 and C327 (4), acting as a built-in counter ion, preventing backtransfer of the released ion, and allowing completion of the reaction cycle.

Collectively, the first structure of a P_IB-4_-type ATPase reveals the topology of P_IB-4_-ATPases, displaying an eight helix M-domain configuration, and likely no HMBDs, at least in members without extended N-termini. Major findings include the observation of an ion-release pathway similar as in the related P_IB-2_-ATPases, a previously not observed counterion principle for P-type ATPases, and a unique potassium-independent regulation of the P_IB_-transport cycle (Figure 4). Thus, our results significantly increase the understanding of heavy-metal homeostasis in cells. The novel identified putative inhibitors and the partially overlapping mechanistic principles of P_IB-2_- and P_IB-4_-ATPases also open up a novel avenue for development of compounds accessible from outside the cell against these P_IB_ groups, to combat global threats such as multidrug resistance and/or tuberculosis or for biotechnological purposes.

## Materials and methods

**Key resources table:** 

Reagent type (species) or resource	Designation	Source or reference	Identifiers	Additional information
Gene (*Sulfitobacter* sp. (strain NAS-14.1))	NAS141_02821	Synthetic	Uniprot: A3T2G5	
Cell line (*Escherichia coli*)	C41(DE3)	Sigma-Aldrich		Chemically competent cells
Cell line(*Mycobacterium bovis*)	BCG Montreal		ATCC 35735	
Software, algorithm	Phenix		RRID:SCR_014224	https://www.phenix-online.org/
Software, algorithm	ISOLDE	https://doi.org/10.1107/S2059798318002425		https://isolde.cimr.cam.ac.uk/
Software, algorithm	UCSF ChimeraX		RRID:SCR_015872	https://www.cgl.ucsf.edu/chimerax/
Software, algorithm	COOT		RRID:SCR_014222	http://www2.mrc-lmb.cam.ac.uk/personal/pemsley/coot/
Software, algorithm	Pymol		RRID:SCR_000305	http://www.pymol.org/

### Overproduction and purification of sCoaT

Forms of the 72 kDa sCoaT from *Sulfitobacter* sp. NAS14-1 (UniProt ID A3T2G5) were transformed into *E. coli* (C41 strain) cells. The cells were cultured in LB medium at 37°C with shaking at 175 rpm in baffled flasks until the optical density (600 nm) reached 0.6–1, cooled to 18°C, and then induced with 1 mM IPTG for 16 hr. Harvested cells were resuspended in buffer A (1 g cells per 5 ml buffer) containing 20 mM Tris–HCl, pH = 7.6, 200 mM KCl, 20% (vol/vol) glycerol and frozen at −80°C until further use. Cells were disrupted by two runs in a high-pressure homogenizer (Constant System) at 25,000 psi following addition of 5 mM of fresh β-mercaptoethanol (BME), 5 mM MgCl_2_, 1 mM phenylmethanesulphonyl fluoride, 2 μg/ml DNase I and Roche protease inhibitor cocktail (1 tablet for 6 l cells). The sample was kept at 4°C throughout the purification. Cellular debris was pelleted via centrifugation at 20,000 × *g* for 20 min. Membranes were isolated by ultracentrifugation for 3 hr at 185,500× *g*, and resuspended in 10 ml buffer B (20 mM Tris–HCl, pH = 7.6, 200 mM KCl, 1 mM MgCl_2_, 5 mM BME, and 20% [vol/vol] glycerol) per g membranes and frozen at −80°C until further use. The protein concentration in the membranes was estimated using the Bradford, 1976 assay. Proteins were solubilized through supplementation of 1% (wt/vol) final concentration *n*-dodecyl-β-D-maltopyranoside (DDM) and 3 mg/ml final total protein concentration in buffer B with gentle stirring for 2 hr. Unsolubilized material was removed by ultracentrifugation for 1 hr at 185,500 × *g*. The supernatant was supplemented with imidazole to a final concentration of 30 mM and solid KCl (500 mM final concentration), filtered (0.22 mm), and then applied to 5 ml HiTrap Chelating HP columns (GE Healthcare, protein from 6 l cells per column) charged with Ni^2+^ and equilibrated with four column volumes of buffer C (20 mM Tris–HCl, pH = 7.6, 200 mM KCl, 1 mM MgCl_2_, 5 mM BME, 150 mg/ml octaethylene glycol monododecyl ether [C_12_E_8_] and 20% [vol/vol] glycerol). Proteins were eluted using a gradient, ending with buffer C containing 500 mM imidazole. Eluted protein was assessed using sodium dodecyl sulphate–polyacrylamide gel electrophoresis (SDS–PAGE), and the fractions containing sCoaT concentrated to approximately 20 mg/ml using VivaSpin concentrators (MWCO = 50 kDa). 10 mg concentrated protein was subjected to size-exclusion chromatography using a Superose six gel-filtration column (GE-Healthcare), pre-equilibrated with 50 ml buffer E (20 mM Tris–HCl, pH = 7.6, 80 mM KCl, 1 mM MgCl_2_, 5 mM BME, 150 mg/ml C_12_E_8_ and 20% [vol/vol] glycerol). Fractions containing purified sCoaT were pooled, and concentrated to approximately 10 mg/ml, flash frozen in liquid nitrogen, and stored at −80°C until further use. For the experiments to assess K^+^ dependence, the buffer E was replaced with 20 mM Tris–HCl, pH = 7.5, 1 mM MgCl_2_, 5 mM BME, 0.15 mg/ml C_12_E_8_, and 20% (vol/vol) glycerol.

### Crystallization

10 mg/ml sCoaT was supplemented with 3 mg/ml (final concentration) DOPC and 6 mg/ml (final concentration) C_12_E_8_, incubated at 4°C and stirring for 16–48 hr (modified HiLiDe method Gourdon et al., 2011a). Aggregates and insoluble DOPC were then removed by ultracentrifugation at 50,000 ×*g* for 10 min. 2 mM AlCl_3_ or BeSO_4_, 10 mM NaF, and 2 mM EGTA (final concentrations) were supplemented and incubated on ice for 30 min. Crystals were grown using the hanging drop vapour diffusion method at 19°C. E2-AlF_4_^−^ crystals were grown with a reservoir solution containing 200 mM MgCl_2_, 14% (vol/vol) PEG1500, 10 mM tris(2-carboxyethyl)phosphine, 10% (vol/vol) glycerol, 3% 2-methyl-2,4-pentanediol, and 100 mM sodium acetate, pH = 5.0. The E2-BeF_3_^−^ crystals were grown with a reservoir solution containing 200 mM magnesium formate, 14% (vol/vol) PEG5000, 100 mM sodium acetate, pH = 4.0, and 0.5% (vol/vol) 2-propanol was added as an additive. Crystals were fished using litholoops (Molecular Dimensions), flash cooled in liquid nitrogen, and tested at synchrotron sources. Complete final data sets were collected at the Swiss Light Source, the Paul Scherrer Institute, Villigen, beam line X06SA.

### Structure determination and refinement

Collected data were processed and scaled with XDS (Table 1). For the E2-AlF_4_^−^ structure, initial phases were obtained by the MR method using software PHASER (McCoy et al., 2007) of the Phenix package (Liebschner et al., 2019), and using the AlF_4_^−^-stabilized structure of SsZntA (PDB ID: 4UMW) as a search model. The E2-BeF_3_^−^ structure was solved using the generated E2-AlF_4_^−^ structure as a MR model. Both crystal forms display poor crystal packing between the membrane domains (Figure 1—figure supplement 2), deteriorating the quality of the electron density maps in these regions (Figure 1—figure supplement 3). In this light, model building of the membrane domains was executed with particular prudence, taking into consideration the connectivity to the well-resolved soluble domains, distinct structural features as well as sequence and structure conservation patterns. Examples of such include the conserved GG motif that forms the kink in MB helix, which is clearly identified also at low length, the SPC motif that twists the M4 helix and the conserved and functionally important well-resolved residue H657 that assisted assigning nearby residues.

Initial manual model building was performed primarily using COOT (Emsley et al., 2010). ISOLDE (Croll, 2018) in ChimeraX (Goddard et al., 2018) was employed for model building and analysis, and was critical for obtainment of the final models with reasonable chemical restraints and low clash score. In particular, ISOLDE’s interactive register shifting tool was instrumental in determining the register of the most weakly resolved TM helices. Secondary structure restraints were applied in some flexible regions, also taking into consideration homology to sCoaT and other models.

During final refinements with phenix.refine (Afonine et al., 2012), the geometry was restrained in torsion space to ISOLDE’s output. Molprobity was exploited for structure validation (Williams et al., 2018). The final models are lacking the first 40 residues only, which is shorter than a classical MBD of 67 amino acids. All structural figures were generated using Pymol (DeLano, 2002). Statistics for the final models were 96.70, 3.30, 0.20, and 0,74 for E2-BeF_3_^−^ and 93.24, 6.13, 0.63, and 8.31 for E2-AlF_4_^−^ in Ramachandran favored and allowed regions, and for rotamer outliers and clash score, respectively.

Activity assay sCoaT forms were functionally characterized using the Baginski method to assess the amount of released inorganic phosphate (Baginski et al., 1967). Briefly, 0.5 μg of purified sCoaT mixed with reaction buffer containing 40 mM MOPS–KOH, pH = 6.8, 5 mM KCl, 5 mM MgCl_2_, 150 mM NaCl, 0.3 mg/ml C_12_E_8_, 0.12 mg/ml soybean lipid, 5 mM NaN_3_, and 0.25 mM Na_2_MoO_4_ in a total volume of 50 μl. For metal stimulation assays, different heavy-metal ions or EGTA were supplemented the reaction buffer to a final concentration of 50 µM. For inhibitor screening (see how inhibitors were identified below), different concentrations of inhibitors were added to the reaction buffer containing 50 μM ZnCl_2_. The samples were then incubated at 37°C with 500 rpm shaking for 5 min, and then supplemented with 5 mM ATP (final concentration) to start the reaction, and incubated at 37°C with 1000 rpm shaking for 10 min. 50 μl freshly prepared stop solution containing 2.5% (wt/vol) ascorbic acid, 0.4 M (vol/vol) HCl, and 1% SDS was then supplemented to stop the reaction and start colour development. 75 μl colour solution (2% [wt/vol] arsenite, 2% [vol/vol] acetic acid, and 3.5% [wt/vol] sodium citrate) was added to the mixture following 10-min incubation at room temperature. Absorbance was measured at 860 nm after another 30-min incubation at room temperature. For the experiments to assess K^+^ dependence, the reaction buffer was replaced with 40 mM Tris–HCl, pH = 7.5, 5 mM MgCl_2_, 3.0 mg/ml C_12_E_8_, and 1.2 mg/ml soybean lipid in a total volume of 50 μl.

### Inhibitor screening

The inhibitor screening experiments were initially carried out on the zinc transporting P_IB-2_-type ATPase ZntA from *Shigella sonnei* (SsZntA). SsZntA was produced and purified as described previously (Wang et al., 2014) and the inhibitory effect of approximately 20,000 compounds was assessed by the Chemical biology Consortium Sweden (CBCS). Briefly, the ATPase activity of 0.7 µM highly pure protein was measured in the presence of 50 µM inhibitor through the release of inorganic phosphate (P_i_) by the Baginski assay (Baginski et al., 1967) in a total volume of 200 nl as reported earlier (Wang et al., 2014). The inorganic phosphate was detected with Malachite Green reagent (0.005% Carbinol hydrochloride, 1.7% sulfuric acid, 0.14% ammonium molybdate, 0.025% Triton-X) at an absorbance of 620 nm.

### Minimum inhibitory concentration

*Mycobacterium bovis* bacillus Calmette–Guerin (BCG) Montreal containing the pSMT1-*luxAB* plasmid was prepared as previously described (Snewin et al., 1999). Briefly, the mycobacteria were grown in Middlebrook 7H9 broth, supplemented with 10% ADC enrichment (Middlebrook Albumin Dextrose Catalase Supplement, Becton Dickinson, Oxford, UK) and hygromycin (50 mg/l; Roche, Lewes, UK), the culture was washed twice with sterile PBS, and resuspended in broth and then dispensed into vials. Glycerol was added to a final concentration of 25% and the vials were frozen at −80°C. Prior to each experiment, a vial was defrosted, added to 9 ml of 7H9/ADC/hygromycin medium, and incubated with shaking for 72 hr at 37°C. Mycobacteria were then centrifuged for 10 min at 3000 × *g*, washed twice with PBS, and resuspended in 10 ml of PBS. Resazurin microtiter assay was used to determine the minimum inhibitory concentration (MIC_90_) for the inhibitors against the mycobacterial strain. The inhibitors (10 µl) were added to bacterial suspensions (90 µl) on a 96-well plate at a concentration range between 0.4 and 50.0 µM. MIC was determined by the colour change using resazurin (1:10 vol/vol, PrestoBlue Cell viability reagent, Thermo Scientific). MIC was determined after 1 week by adding 10 µl resazurin followed by incubation overnight, corresponding to 90% inhibition.

### Human cytotoxicity assays

Human venous blood mononuclear cells were obtained from healthy volunteers using a Lymphoprep density gradient (Axis-Shield, Oslo, Norway) according to the manufacturer’s instructions. To obtain pure monocytes, CD14 microbeads were applied to the cell suspension, washed, and passed through a LS column according to the manufacturer’s description (130-050-201, 130-042-401, Miltenyi Biotec, USA). The monocytes were counted (Sysmex), diluted in RPMI 1640 supplemented with 5% FCS, NEAA, 1 mM sodium pyruvate, 0.1 mg/ml gentamicin (11140-035, 111360-039, 15710-49, Gibco, Life Technologies) and 50 ng/ml GM-CSF (215 GM, R&D Systems) and seeded in 96-well plates (10^5^/well) for a week to differentiate into macrophages. Infection experiments were performed in RPMI 1640 without Gentamicin. The medium was replaced with fresh medium containing 6.3, 12.5, 25, or 50 μM inhibitor or DMSO and incubated 24 hr in 5% CO_2_ atmosphere. For cytotoxicity measurement, 10 μl 3-(4,5-dimethylthiazol-2-yl)–2,5 diphenyltetrazolium bromide solution (Sigma) was added to each well according to the manufacturer’s instructions and analysed in a spectrophotometer at 580 nm. NZX cytotoxicity was further examined by ATPlite assays. Primary macrophages were treated with 6.3, 12.5, 25, or 50 μM inhibitor or DMSO (Sigma) for 24 hr. Cell viability was assessed with cellular ATP levels using ATPlite kit (6016943, Perkin Elmer) compared to untreated controls, according to the manufacturer’s instructions.

### MD simulation

The two crystal structures, E2-AlF_4_^−^ and E2-BeF_3_^−^, were inserted into a DOPC (1,2-dioleoyl-sn-glycero-3-phosphocholine) membrane patch using the CHARMM-GUI membrane builder (Wu et al., 2014). The membrane positions were predicted by the Orientations of Proteins in Membranes (OPM) server (Lomize et al., 2012). During the simulation equilibration phase, position restraints were gradually released from the water and lipids for a total of 30 ns followed by 500 ns non-restrained production runs. Each protein state was simulated in independent repeat simulations starting from a different set of initial velocities, adding up to a sampling total of 500 ns × 4. A Nose–Hoover temperature coupling (Nosé and Klein, 2006) was applied using a reference temperature of 310 K. A Parrinello–Rahman pressure coupling (Parrinello and Rahman, 1981) was applied with a reference pressure of 1 bar and compressibility of 4.5e−5 bar^−1^ in a semi-isotropic environment. The TIP3P water model was used and the system contained 0.15 M NaCl. The E2-AlF_4_^−^ system was composed of 256 lipids and 29,429 water molecules while E2-BeF_3_^−^ system was composed of 254 lipids and 30,535 water molecules. The systems were equilibrated and simulated using the GROMACS-2021 simulation package (Abraham et al., 2015) and CHARMM36 all-atom force fields (Best et al., 2012) for the protein and lipids. The membrane domain was used as alignment reference for the root means square deviation and centre-of-mass calculations, and the protein backbone was used as alignment reference for calculating the root mean square fluctuation. The secondary structure was assessed with the do_dssp tool in GROMACS-2021 (Abraham et al., 2015).

Atomic coordinates and structure factors for the sCoaT AlF_4_^−^- and BeF_3_^−^-stabilized crystal structures have been deposited at the Protein Data Bank (PDB) under accession codes 7QBZ and 7QC0. The authors declare no competing financial interests. Correspondence and requests for materials should be addressed to P.G. (pontus@sund.ku.dk).

## Data Availability

Atomic coordinates and structure factors for the sCoaT AlF4- and BeF3-stabilized crystal structures have been deposited at the Protein Data Bank (PDB) under accession codes 7QBZ and 7QC0. The following datasets were generated: GourdonP GrønbergC HuQ MahatoDR LonghinE SalustrosN DuelliA LyuP ErikssonJ RaoKU HendersonDI MeloniG AnderssonM CrollT GodalyG WangK BågenholmV 2022Structure and ion-release mechanism of PIB-4-type ATPasesRCSB Protein Data Bank7QBZ10.7554/eLife.73124PMC888099734951590 GourdonP GrønbergC HuQ MahatoDR LonghinE SalustrosN DuelliA LyuP BågenholmV ErikssonJ RaoKU HendersonDI MeloniG AnderssonM CrollT GodalyG WangW 2022Structure and ion-release mechanism of PIB-4-type ATPasesRCSB Protein Data Bank7QC010.7554/eLife.73124PMC888099734951590

## Additional files

Transparent reporting form

## References

[bib1] Abe K, Irie K, Nakanishi H, Suzuki H, Fujiyoshi Y (2018). Crystal structures of the gastric proton pump. Nature.

[bib2] Abeyrathna N, Abeyrathna S, Morgan MT, Fahrni CJ, Meloni G, Cu T (2020). Transmembrane Cu(I) P-type ATPase pumps are electrogenic uniporters. Dalton Transactions.

[bib3] Abraham MJ, Murtola T, Schulz R, Páll S, Smith JC, Hess B, Lindahl E (2015). GROMACS: High performance molecular simulations through multi-level parallelism from laptops to supercomputers. SoftwareX.

[bib4] Afonine PV, Grosse-Kunstleve RW, Echols N, Headd JJ, Moriarty NW, Mustyakimov M, Terwilliger TC, Urzhumtsev A, Zwart PH, Adams PD (2012). Towards automated crystallographic structure refinement with phenix.refine. Acta Crystallographica. Section D, Biological Crystallography.

[bib5] Albers RW, Fahn S, Koval GJ (1963). The role of sodium ions in the activation of electrophorus electric organ adenosine triphosphatase. PNAS.

[bib6] Andersson M, Mattle D, Sitsel O, Klymchuk T, Nielsen AM, Møller LB, White SH, Nissen P, Gourdon P (2014). Copper-transporting P-type ATPases use a unique ion-release pathway. Nature Structural & Molecular Biology.

[bib7] Argüello JM (2003). Identification of ion-selectivity determinants in heavy-metal transport P1B-type ATPases. The Journal of Membrane Biology.

[bib8] Baginski ES, Foà PP, Zak B (1967). Microdetermination of inorganic phosphate, phospholipids, and total phosphate in biologic materials. Clinical Chemistry.

[bib9] Best RB, Zhu X, Shim J, Lopes PEM, Mittal J, Feig M, Mackerell AD (2012). Optimization of the additive CHARMM all-atom protein force field targeting improved sampling of the backbone φ, ψ and side-chain χ(1) and χ(2) dihedral angles. Journal of Chemical Theory and Computation.

[bib10] Borrelly GPM, Rondet SAM, Tottey S, Robinson NJ (2004). Chimeras of P-type ATPases and their transcriptional regulators: contributions of a cytosolic amino-terminal domain to metal specificity. Molecular Microbiology.

[bib11] Botella H, Peyron P, Levillain F, Poincloux R, Poquet Y, Brandli I, Wang C, Tailleux L, Tilleul S, Charrière GM, Waddell SJ, Foti M, Lugo-Villarino G, Gao Q, Maridonneau-Parini I, Butcher PD, Castagnoli PR, Gicquel B, de Chastellier C, Neyrolles O (2011). Mycobacterial p(1)-type ATPases mediate resistance to zinc poisoning in human macrophages. Cell Host & Microbe.

[bib12] Bradford MM (1976). A rapid and sensitive method for the quantitation of microgram quantities of protein utilizing the principle of protein-dye binding. Analytical Biochemistry.

[bib13] Bull PC, Thomas GR, Rommens JM, Forbes JR, Cox DW (1993). The Wilson disease gene is a putative copper transporting P-type ATPase similar to the Menkes gene. Nature Genetics.

[bib14] Croll TI (2018). ISOLDE: a physically realistic environment for model building into low-resolution electron-density maps. Acta Crystallographica. Section D, Structural Biology.

[bib15] DeLano WL (2002). Pymol: An open-source molecular graphics tool. CCP4 Newsletter on Protein Crystallography.

[bib16] Drees SL, Beyer DF, Lenders-Lomscher C, Lübben M (2015). Distinct functions of serial metal-binding domains in the *Escherichia coli* P1 B -ATPase CopA. Molecular Microbiology.

[bib17] Dyla M, Kjærgaard M, Poulsen H, Nissen P (2020). Structure and Mechanism of P-Type ATPase Ion Pumps. Annual Review of Biochemistry.

[bib18] Emsley P, Lohkamp B, Scott WG, Cowtan K (2010). Features and development of Coot. Acta Crystallographica. Section D, Biological Crystallography.

[bib19] Faxén K, Andersen JL, Gourdon P, Fedosova N, Morth JP, Nissen P, Møller JV (2011). Characterization of a Listeria monocytogenes Ca(2+) pump: a SERCA-type ATPase with only one Ca(2+)-binding site. The Journal of Biological Chemistry.

[bib20] Goddard TD, Huang CC, Meng EC, Pettersen EF, Couch GS, Morris JH, Ferrin TE (2018). UCSF ChimeraX: Meeting modern challenges in visualization and analysis. Protein Science.

[bib21] González-Guerrero M, Argüello JM (2008). Mechanism of Cu+-transporting ATPases: soluble Cu+ chaperones directly transfer Cu+ to transmembrane transport sites. PNAS.

[bib22] Gourdon P, Andersen JL, Hein KL, Bublitz M, Pedersen BP, Liu XY, Yatime L, Nyblom M, Nielsen TT, Olesen C, Møller JV, Nissen P, Morth JP (2011a). HiLiDe—Systematic Approach to Membrane Protein Crystallization in Lipid and Detergent. Crystal Growth & Design.

[bib23] Gourdon P, Liu XY, Skjørringe T, Morth JP, Møller LB, Pedersen BP, Nissen P (2011b). Crystal structure of a copper-transporting PIB-type ATPase. Nature.

[bib24] Hansen SB (2020). The Crystal Structure of the Ca2+-ATPase 1 from Listeria Monocytogenes Reveals a Pump Primed for Dephosphorylation. bioRxiv.

[bib25] Joshi SM, Pandey AK, Capite N, Fortune SM, Rubin EJ, Sassetti CM (2006). Characterization of mycobacterial virulence genes through genetic interaction mapping. PNAS.

[bib26] Kozlowski H, Janicka-Klos A, Brasun J, Gaggelli E, Valensin D, Valensin G (2009). Copper, iron, and zinc ions homeostasis and their role in neurodegenerative disorders (metal uptake, transport, distribution and regulation). Coordination Chemistry Reviews.

[bib27] Lanzetta PA, Alvarez LJ, Reinach PS, Candia OA (1979). An improved assay for nanomole amounts of inorganic phosphate. Analytical Biochemistry.

[bib28] Liebschner D, Afonine PV, Baker ML, Bunkóczi G, Chen VB, Croll TI, Hintze B, Hung LW, Jain S, McCoy AJ, Moriarty NW, Oeffner RD, Poon BK, Prisant MG, Read RJ, Richardson JS, Richardson DC, Sammito MD, Sobolev OV, Stockwell DH, Terwilliger TC, Urzhumtsev AG, Videau LL, Williams CJ, Adams PD (2019). Macromolecular structure determination using X-rays, neutrons and electrons: recent developments in Phenix. Acta Crystallographica. Section D, Structural Biology.

[bib29] Lomize MA, Pogozheva ID, Joo H, Mosberg HI, Lomize AL (2012). OPM database and PPM web server: resources for positioning of proteins in membranes. Nucleic Acids Research.

[bib30] Mattle D, Sitsel O, Autzen HE, Meloni G, Gourdon P, Nissen P (2013). On allosteric modulation of P-type Cu(+)-ATPases. Journal of Molecular Biology.

[bib31] Mattle D, Zhang L, Sitsel O, Pedersen LT, Moncelli MR, Tadini-Buoninsegni F, Gourdon P, Rees DC, Nissen P, Meloni G (2015). A sulfur-based transport pathway in Cu+-ATPases. EMBO Reports.

[bib32] McCoy AJ, Grosse-Kunstleve RW, Adams PD, Winn MD, Storoni LC, Read RJ (2007). Phaser crystallographic software. Journal of Applied Crystallography.

[bib33] Mitra B, Sharma R (2001). The cysteine-rich amino-terminal domain of ZntA, a Pb(II)/Zn(II)/Cd(II)-translocating ATPase from *Escherichia coli*, is not essential for its function. Biochemistry.

[bib34] Moller JV, Olesen C, Winther AML, Nissen P (2010). The sarcoplasmic Ca2+-ATPase: design of a perfect chemi-osmotic pump. Quarterly Reviews of Biophysics.

[bib35] Moreno I, Norambuena L, Maturana D, Toro M, Vergara C, Orellana A, Zurita-Silva A, Ordenes VR (2008). AtHMA1 is a thapsigargin-sensitive Ca2+/heavy metal pump. The Journal of Biological Chemistry.

[bib36] Morin I, Gudin S, Mintz E, Cuillel M (2009). Dissecting the role of the N-terminal metal-binding domains in activating the yeast copper ATPase in vivo. The FEBS Journal.

[bib37] Morth JP, Pedersen BP, Toustrup-Jensen MS, Sørensen TL-M, Petersen J, Andersen JP, Vilsen B, Nissen P (2007). Crystal structure of the sodium-potassium pump. Nature.

[bib38] Nakanishi H, Nishizawa T, Segawa K, Nureki O, Fujiyoshi Y, Nagata S, Abe K (2020). Transport Cycle of Plasma Membrane Flippase ATP11C by Cryo-EM. Cell Reports.

[bib39] Nosé S, Klein ML (2006). Constant pressure molecular dynamics for molecular systems. Molecular Physics.

[bib40] Olesen C, Sørensen TL-M, Nielsen RC, Møller JV, Nissen P (2004). Dephosphorylation of the calcium pump coupled to counterion occlusion. Science.

[bib41] Olesen C, Picard M, Winther A-ML, Gyrup C, Morth JP, Oxvig C, Møller JV, Nissen P (2007). The structural basis of calcium transport by the calcium pump. Nature.

[bib42] Osman D, Martini MA, Foster AW, Chen J, Scott AJP, Morton RJ, Steed JW, Lurie-Luke E, Huggins TG, Lawrence AD, Deery E, Warren MJ, Chivers PT, Robinson NJ (2019). Bacterial sensors define intracellular free energies for correct enzyme metalation. Nature Chemical Biology.

[bib43] Parrinello M, Rahman A (1981). Polymorphic transitions in single crystals: A new molecular dynamics method. Journal of Applied Physics.

[bib44] Patel SJ, Lewis BE, Long JE, Nambi S, Sassetti CM, Stemmler TL, Argüello JM (2016). Fine-tuning of Substrate Affinity Leads to Alternative Roles of Mycobacterium tuberculosis Fe2+-ATPases. The Journal of Biological Chemistry.

[bib45] Pedersen BP, Buch-Pedersen MJ, Morth JP, Palmgren MG, Nissen P (2007). Crystal structure of the plasma membrane proton pump. Nature.

[bib46] Pi H, Patel SJ, Argüello JM, Helmann JD (2016). The Listeria monocytogenes Fur-regulated virulence protein FrvA is an Fe(II) efflux P1B4 -type ATPase. Molecular Microbiology.

[bib47] Post RL, Sen AK (1965). An enzymatic mechanism of active sodium and potassium transport. The Journal of Histochemistry and Cytochemistry.

[bib48] Raimunda D, Long JE, Sassetti CM, Argüello JM (2012). transporting P(1B4)-ATPase of Mycobacterium smegmatis. Molecular Microbiology.

[bib49] Rosenzweig AC, Argüello JM (2012). Toward a molecular understanding of metal transport by P(1B)-type ATPases. Current Topics in Membranes.

[bib50] Sassetti CM, Rubin EJ (2003). Genetic requirements for mycobacterial survival during infection. PNAS.

[bib51] Schack VR, Morth JP, Toustrup-Jensen MS, Anthonisen AN, Nissen P, Andersen JP, Vilsen B (2008). Identification and function of a cytoplasmic K+ site of the Na+, K+ -ATPase. The Journal of Biological Chemistry.

[bib52] Scherer J, Nies DH (2009). CzcP is a novel efflux system contributing to transition metal resistance in Cupriavidus metallidurans CH34. Molecular Microbiology.

[bib53] Seigneurin-Berny D, Gravot A, Auroy P, Mazard C, Kraut A, Finazzi G, Grunwald D, Rappaport F, Vavasseur A, Joyard J, Richaud P, Rolland N (2006). HMA1, a new Cu-ATPase of the chloroplast envelope, is essential for growth under adverse light conditions. The Journal of Biological Chemistry.

[bib54] Shinoda T, Ogawa H, Cornelius F, Toyoshima C (2009). Crystal structure of the sodium-potassium pump at 2.4 A resolution. Nature.

[bib55] Sitsel O, Grønberg C, Autzen HE, Wang K, Meloni G, Nissen P, Gourdon P (2015). Structure and Function of Cu(I)- and Zn(II)-ATPases. Biochemistry.

[bib56] Smith AT, Smith KP, Rosenzweig AC (2014). Diversity of the metal-transporting P1B-type ATPases. Journal of Biological Inorganic Chemistry.

[bib57] Smith AT, Ross MO, Hoffman BM, Rosenzweig AC (2017). Metal Selectivity of a Cd-, Co-, and Zn-Transporting P1B-type ATPase. Biochemistry.

[bib58] Snewin VA, Gares MP, Gaora PO, Hasan Z, Brown IN, Young DB (1999). Assessment of immunity to mycobacterial infection with luciferase reporter constructs. Infection and Immunity.

[bib59] Sørensen TL-M, Clausen JD, Jensen A-ML, Vilsen B, Møller JV, Andersen JP, Nissen P (2004). Localization of a K+ -binding site involved in dephosphorylation of the sarcoplasmic reticulum Ca2+ -ATPase. The Journal of Biological Chemistry.

[bib60] Sørensen TL-M, Olesen C, Jensen A-ML, Møller JV, Nissen P (2006). Crystals of sarcoplasmic reticulum Ca2+-ATPase. Journal of Biotechnology.

[bib61] Toyoshima C, Nakasako M, Nomura H, Ogawa H (2000). Crystal structure of the calcium pump of sarcoplasmic reticulum at 2.6 A resolution. Nature.

[bib62] Toyoshima C, Nomura H (2002). Structural changes in the calcium pump accompanying the dissociation of calcium. Nature.

[bib63] Toyoshima C, Nomura H, Tsuda T (2004). Lumenal gating mechanism revealed in calcium pump crystal structures with phosphate analogues. Nature.

[bib64] Toyoshima C, Iwasawa S, Ogawa H, Hirata A, Tsueda J, Inesi G (2013). Crystal structures of the calcium pump and sarcolipin in the Mg2+-bound E1 state. Nature.

[bib65] Vulpe C, Levinson B, Whitney S, Packman S, Gitschier J (1993). Isolation of a candidate gene for Menkes disease and evidence that it encodes a copper-transporting ATPase. Nature Genetics.

[bib66] Waldron KJ, Rutherford JC, Ford D, Robinson NJ (2009). Metalloproteins and metal sensing. Nature.

[bib67] Wang K, Sitsel O, Meloni G, Autzen HE, Andersson M, Klymchuk T, Nielsen AM, Rees DC, Nissen P, Gourdon P (2014). Structure and mechanism of Zn2+-transporting P-type ATPases. Nature.

[bib68] Williams CJ, Headd JJ, Moriarty NW, Prisant MG, Videau LL, Deis LN, Verma V, Keedy DA, Hintze BJ, Chen VB, Jain S, Lewis SM, Arendall WB, Snoeyink J, Adams PD, Lovell SC, Richardson JS, Richardson DC (2018). MolProbity: More and better reference data for improved all-atom structure validation. Protein Science.

[bib69] Winther A-ML, Bublitz M, Karlsen JL, Møller JV, Hansen JB, Nissen P, Buch-Pedersen MJ (2013). The sarcolipin-bound calcium pump stabilizes calcium sites exposed to the cytoplasm. Nature.

[bib70] Wu EL, Cheng X, Jo S, Rui H, Song KC, Dávila-Contreras EM, Qi Y, Lee J, Monje-Galvan V, Venable RM, Klauda JB, Im W (2014). CHARMM-GUI Membrane Builder toward realistic biological membrane simulations. Journal of Computational Chemistry.

[bib71] Yu CH, Yang N, Bothe J, Tonelli M, Nokhrin S, Dolgova NV, Braiterman L, Lutsenko S, Dmitriev OY (2017). The metal chaperone Atox1 regulates the activity of the human copper transporter ATP7B by modulating domain dynamics. The Journal of Biological Chemistry.

[bib72] Zhitnitsky D, Lewinson O (2014). Identification of functionally important conserved trans-membrane residues of bacterial PIB -type ATPases. Molecular Microbiology.

[bib73] Zielazinski EL, Cutsail GE, Hoffman BM, Stemmler TL, Rosenzweig AC (2012). Characterization of a cobalt-specific P(1B)-ATPase. Biochemistry.

